# The Kohlschütter-Tönz syndrome associated gene Rogdi encodes a novel presynaptic protein

**DOI:** 10.1038/s41598-017-16004-1

**Published:** 2017-11-17

**Authors:** Donatus Riemann, Rebecca Wallrafen, Thomas Dresbach

**Affiliations:** Institute for Anatomy and Embryology, University Medical Centre Göttingen, Kreuzbergring 36, 37075 Göttingen, Germany

## Abstract

Mutations in the human homolog of the Drosophila gene Rogdi cause Kohlschütter-Tönz syndrome. This disorder is characterised by amelogenesis imperfecta, as well as severe neurological symptoms including epilepsy and psychomotor delay. However, little is known about the protein encoded by Rogdi, and hence the pathogenic mechanisms underlying Kohlschütter-Tönz syndrome have remained elusive. Using immunofluorescence of rat cultured hippocampal neurons and brain sections we find that Rogdi is enriched at synaptic sites. In addition, recombinant GFP-Rogdi expressed in cultured neurons was efficiently targeted to presynaptic sites, where it colocalised with the presynaptic scaffolding protein Bassoon and the synaptic vesicle markers Synaptophysin, Synapsin-1, VAMP2/Synaptobrevin and Mover. Our data indicate that GFP-Rogdi harbours efficient signals for presynaptic targeting, and that Rogdi is a presynaptic protein. Thus, the neurological symptoms associated with Kohlschütter-Tönz syndrome may arise from presynaptic dysfunction.

## Introduction

Kohlschütter-Tönz syndrome (KTS, MIM 226750) is a rare autosomal recessive disorder first described in 1974^1^. KTS is characterised by a striking combination of symptoms, including amelogenesis imperfecta, epilepsy and severe psychomotor delay^2,3^. Amelogenesis imperfecta is a condition affecting all teeth that manifests with dysplastic enamel stained in brown and yellow colours. It occurs both as part of KTS and as a non-syndromic condition caused by various gene defects.

The devastating neurological symptoms associated with KTS usually occur within the first two years of life. Epileptic seizures are often resistant to pharmacological treatment. Impaired cognitive and motor function (psychomotor delay and regression) develop most frequently after the onset of epilepsy, but are obvious from the time of birth in some cases. Severe intellectual disability, impaired gross and fine motor skills, and spasticity are further characteristics of the syndrome, which may lead to death in childhood or early adulthood^4^.

No abnormal metabolic features are known for KTS, but mutations in the human homolog of the Drosophila gene Rogdi have recently been shown to cause KTS^4–7^. The gene encodes a 30 kDa protein (287 amino acids in man; two isoforms comprised of 268 and 343 amino acids, respectively, in Drosophila) of unknown function. Regarding subcellular localization, nuclear envelope localization of recombinant Rogdi expressed in HEK293 cells and of endogenous Rogdi detected by immunofluorescence in skin fibroblasts and blood cells were reported^5^. More recently, Rogdi was detected in biochemical fractions enriched for synaptic vesicles^8^.

In view of the prominent and severe neurological symptoms of KTS we sought to test whether Rogdi might be a presynaptic protein. We show here that both endogenous and GFP-tagged recombinant Rogdi display a presynaptic localization pattern, indicating that Rogdi is a novel presynaptic protein.

## Results

### Localisation of Endogenous Rogdi in Cultured Neurons

**Figure 1:**
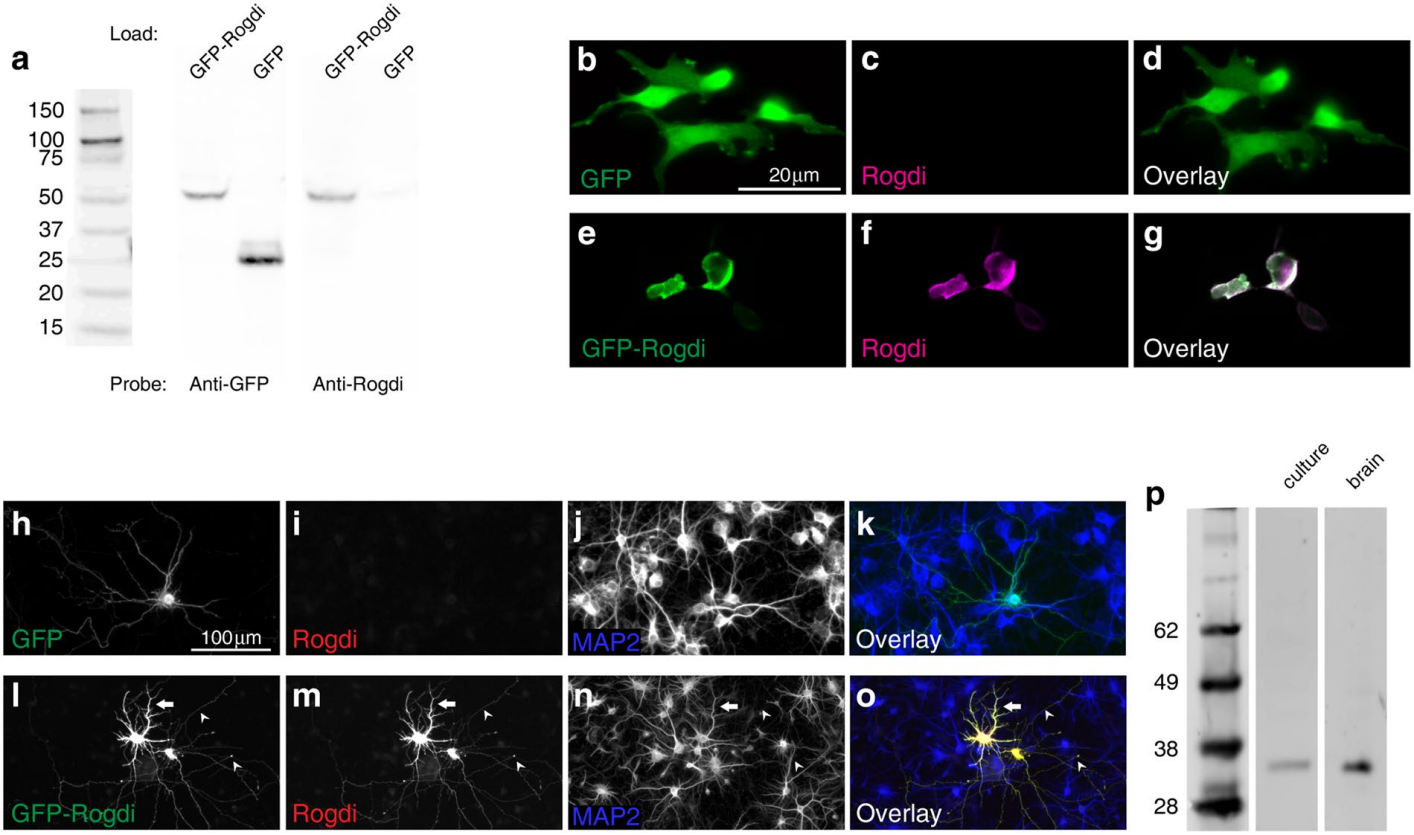
Validation of an Antibody Directed against Amino Acids 14–95 of Human Rogdi. (**a**) Western blot of lysates obtained from transfected HEK293 cells. Lanes were loaded with lysates from cells expressing GFP-Rogdi or GFP. Lanes were probed with an anti-GFP antibody or with the anti-Rogdi antibody. The anti-Rogdi antibody detected GFP-Rogdi, but not GFP, indicating that it detects recombinant Rogdi. (**b**–**g**) Immunofluorescence analysis of HEK293 cells expressing either GFP (**b**–**d**) or GFP-Rogdi (**e**–**g**). The anti-Rogdi antibody detects GFP-Rogdi, but not GFP. (**h**–**o**) Immunofluorescence analysis of cultured hippocampal neurons transfected with Lipofectamine 2000 to express GFP (**h**–**k**) or GFP-Rogdi (**l**–**o**). MAP2 is a marker for the somatodendritic compartment of neurons. MAP2 immunofluorescence indicates the number and density of neurons in the field of view as well as the location of their somatodendritic compartment. The anti-Rogdi antibody does not detect GFP (**h**–**k**), but does detect GFP-Rogdi (**l**–**o**). The field of view displayed in (**l**–**o**) contains two transfected neurons. The antibody detects GFP-Rogdi both in MAP2-positive processes (the arrow points at an example) and in MAP2-negative processes (the arrowheads point at examples), i.e. in all compartments of transfected cells. (**p**) Western Blot of lysate obtained from untransfected DIV10 hippocampal cultures (“culture”) and of rat brain synaptosomes (“brain”). The anti-Rogdi antibody detects a single band. The band corresponds to the expected molecular weight of Rogdi (cropped blot, for original blot see Fig. S3).

To test whether Rogdi might be present at synapses, we aimed to examine the localization of endogenous Rogdi in rat cultured hippocampal neurons by immunofluorescence. To this end, we first tested the specificity of a rabbit polyclonal antibody directed against amino acids 14 through 95 of human Rogdi, where human and rat Rogdi share 95% identity at the amino acid level. This antibody detected recombinant GFP-tagged rat Rogdi, but not GFP, in western blots from transfected HEK293 cells (Fig. 1a). GFP-Rogdi is expected to run as a ca. 50 kDa band, corresponding to 525 amino acids of the fusion protein, while endogenous Rogdi is expected to run at ca. 30 kDa, corresponding to the 287 amino acids of Rogdi. The Rogdi antibody did not detect a 30 kDa band, indicating that Rogdi may be expressed at levels too low for detection or may not be expressed at all in HEK293 cells. This further corroborates that the antibody selectively detects recombinant GFP-Rogdi by western blotting. The antibody also detected GFP-Rogdi, but not GFP, using immunofluorescence of transfected HEK293 cells (Fig. 1b-g). In addition, the antibody detected GFP-Rogdi overexpressed in cultured hippocampal neurons (Fig. 1h-o). This overexpression experiment was designed to test whether the Rogdi antibody detects recombinant Rogdi not only when it is expressed in HEK293 cells but also when it is expressed in cultured neurons. An inherent feature of this approach is that any immunosignal produced by the antibody could represent either recombinant Rogdi or endogenous Rogdi present in cultured neurons. To ascertain that in these experiments the immunosignal represented primarily recombinant Rogdi, we designed a specific protocol: i) we purposely chose a transfection protocol (i.e. liposome-based transfection; see materials and methods) that leads to high protein levels in transfected cells, essentially loading the neurons with GFP-Rogdi; ii) we used immature neurons at day 7 after plating (day 7 *in vitro*; DIV7), assuming that the less mature the neurons are the less endogenous Rogdi might be expressed; iii) we used a 10x objective, yielding a magnification that is too small to detect synaptic immunosignals, if there were any. Under these conditions, the Rogdi antibody did not produce any immunosignal in untransfected cells and in cells expressing GFP (Fig. 1h-k). The antibody did label all compartments of neurons filled with GFP-Rogdi (Fig. 1l-o), including the somatodendritic compartment identified by the marker MAP2, and axons identified as MAP2-negative processes (for a validation of compartment markers, see Figs S1 and S2). In summary, the Rogdi antibody detects recombinant Rogdi both by western blotting and by immunofluorescence. Furthermore, it detected a single band of the expected size for endogenous Rogdi in western blots of untransfected cultured neurons and synaptosomes from adult rat brain (Fig. 1p; to see the original blot, see Fig. S3), suggesting that Rogdi is expressed in neuronal cultures and in the brain. Next, we used this antibody for immunofluorescence analysis of untransfected cultured neurons, to determine the localization of endogenous Rogdi.

**Figure 2:**
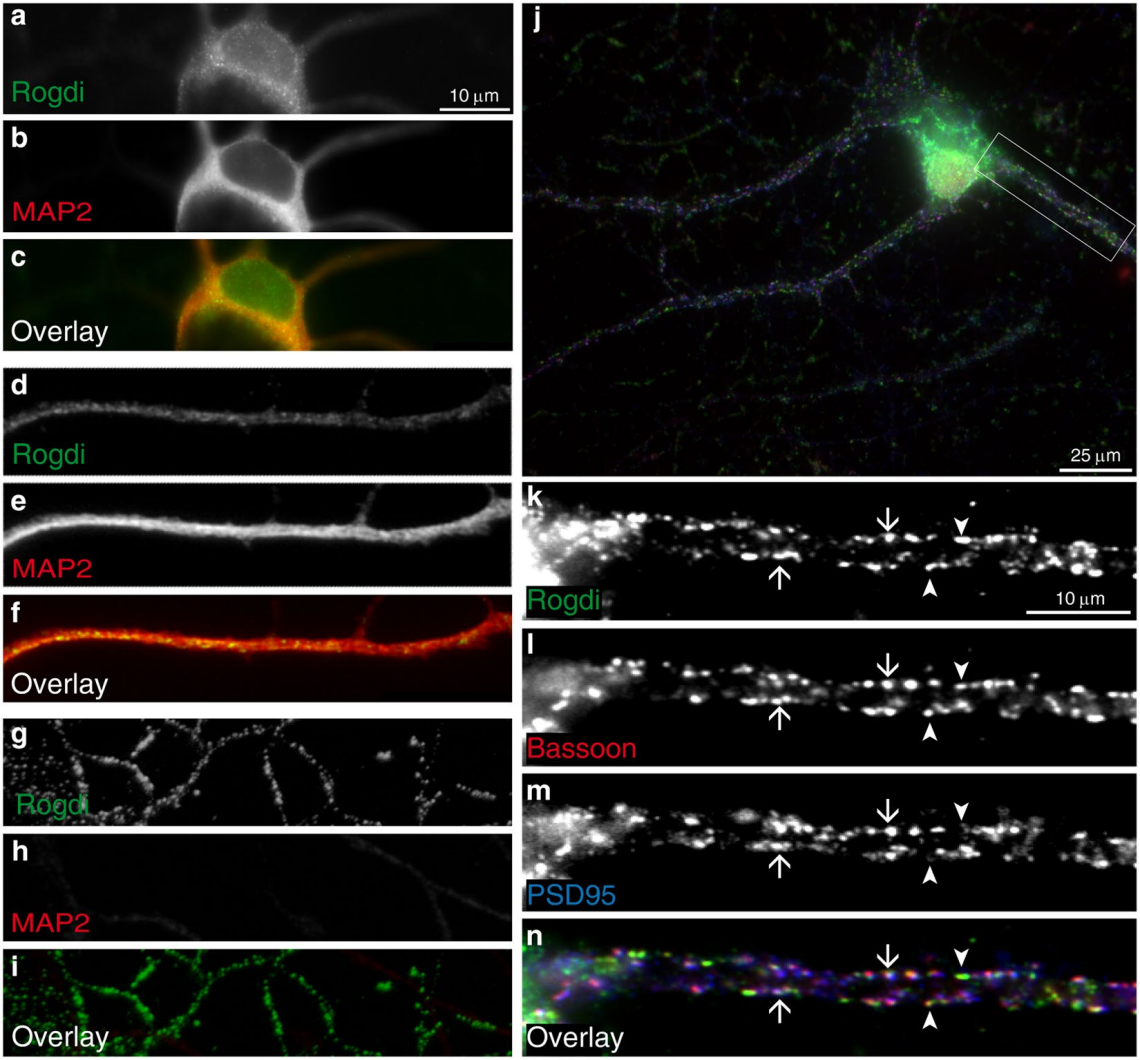
Synaptic Localisation of Endogenous Rogdi in Cultured Hippocampal Neurons. Immunofluorescence localisation of endogenous Rogdi in cultured neurons at an early stage of maturation, i.e. DIV 7 (**a**–**i**) and at a late stage of maturation, i.e. DIV 21 (**j**–**n**). In immature cultures (**a**–**i**), immunofluorescence signals for Rogdi are detected in the soma. Compared to the soma, fluorescence intensity is decreased in the nucleus (**a**–**c**). The soma including the nucleus is in focus, the proximal dendrites are out of focus. The round MAP2-negative structure is the nucleus. Rogdi immunosignals are also detected in dendrites, identified as MAP2 positive processes (**d**–**f**), and in axons, identified as MAP2 negative processes (**g**–**j**). In each compartment, the Rogdi immunofluorescence is homogeneously distributed. In mature cultures (**j**–**n**), Rogdi immunofluorescence manifests as punctate immunosignals lined up along the periphery of dendrites. The size and distribution of these immunosignals resembles the immunosignals of synaptic markers Bassoon (**l**) and PSD95 (**m**). Bassoon is a marker for all synapses, PSD95 is a marker for excitatory synapses. Arrows show examples of colocalisation of Rogdi with Bassoon and PSD95, indicating that Rogdi is present at excitatory synapses. Arrowheads show examples of colocalisation of Rogdi with Bassoon at sites that do not contain PSD95, potentially inhibitory synapses. The soma and nucleus are out of focus in (**j**).

In immature neurons (DIV7), the antibody yielded an immunosignal in the somatodendritic region, identified as parts of the cell positive for the marker MAP2 (Fig. 2a–f), and in axons, identified as MAP2-negative processes (Fig. 2g–i). In the somatodendritic region, Rogdi immunofluorescence was widely and homogeneously distributed, but spared the nucleus. In all regions, including the soma, dendrites and axons, Rogdi immunofluorescence was somewhat granular. Note that the axonal areas in Fig. 2g are likely non-synaptic, since they are not in contact with MAP2-positive processes, i.e. dendrites. This is consistent with the young age (DIV7) of these cultures, where axon-dendrite contacts and synapses are rare. Cultures are considered immature in the first week after plating (i.e. until ca. DIV7) and increasingly mature after DIV10^8^.

To test whether Rogdi is present at synapses, we co-stained for Rogdi, Bassoon and PSD95 in mature neurons (DIV21). Bassoon is a presynaptic active zone protein present at excitatory and inhibitory synapses. PSD95 is a postsynaptic density protein specific for excitatory synapses. All synapses should thus contain Bassoon, while excitatory synapses should contain PSD95 in addition. A striking difference to DIV7 was that at DIV21 Rogdi immunosignals were primarily found in the periphery of dendrites, rather than inside dendrites (Fig. 2j). Higher magnification revealed punctate immunosignals for Rogdi along the periphery of dendrites (Fig. 2k). This is consistent with localization of Rogdi to synapses in mature cultures. Indeed, the punctate immunosignals showed extensive colocalisation with Bassoon, indicating their synaptic localization (Fig. 2l). In addition, we observed Bassoon puncta negative for Rogdi, suggesting that there are active zones with no or with low levels of Rogdi. There were two categories of synaptic Rogdi-puncta: a) some colocalised with Bassoon and PSD95, indicating their localisation at excitatory synapses; b) some colocalised with Bassoon only, presumably reflecting their association with inhibitory synapses. To further corroborate that Rogdi is detected at synapses in cultured neurons, we used yet another synapse marker, the synaptic vesicle protein Synapsin-1, and stained axons using an axon-specific marker called Neurofilament-H (for validation of this marker, see Fig. S2). Interpreting axonal stainings is notoriously difficult, because axons of cultured hippocampal neurons are several millimetres long and form a dense neuropil all over the coverslip. Nonetheless, at sites of the coverslip where the neuropil was loose enough to allow for the detection of parts of individual axons, punctate immunofluorescence for Rogdi was detected within Neurofilament-H positive processes, and these puncta colocalised with the synaptic vesicle marker Synapsin-1 (Fig. S4). We conclude that Rogdi is detected at synapses in mature cultured neurons. To further corroborate this we used a different antibody, raised against full length human Rogdi and validated by knockdown of Rogdi^10^. This antibody detected overexpressed Rogdi in cultured neurons (Fig. S5a-h). When applied to untransfected DIV15 cultures, it yielded a homogeneous staining in the soma, sparing the nucleus, and punctate immunosignals in the periphery of dendrites that showed extensive colocalisation with Bassoon (Fig. S5i-r). Thus, two distinct antisera yielded a similar staining pattern, including colocalisation of punctate Rogdi immunosignals with Bassoon. Overall, these data indicate that in cultured neurons, Rogdi is present at least at a subset of synapses.

### Localisation of Endogenous Rogdi in Brain Sections

**Figure 3:**
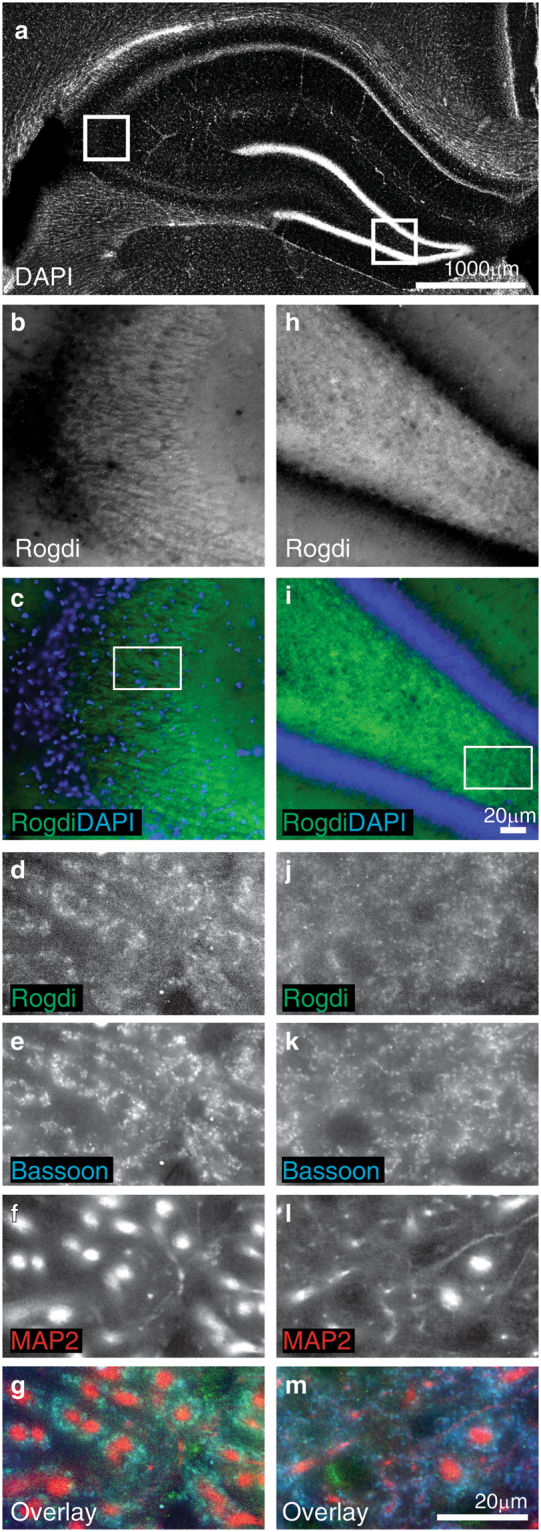
Synaptic Localisation of Endogenous Rogdi in Hippocampal Brain Sections. (**a**) Overview of a DAPI stained brain section indicating where high magnification images were taken (white boxes). The left box includes a part of the hippocampal CA3 region, magnified in (**b**) and (**c**). The right box includes a part of the hilar region of the dentate gyrus, magnified in (**h**) and (**i**). Rogdi immunofluorescence represents a characteristic synaptic pattern: In the CA3 region, Rogdi immunofluorescence is particularly high in the stratum lucidum, where mossy fibres terminate (**b**,**c**). The box in (**c**) is magnified in (**d**–**g**). In the stratum lucidum, Rogdi immunofluorescence surrounds MAP2-labelled dendrites and colocalises with the presynaptic marker Bassoon (**d**–**g**). In the dentate gyrus, Rogdi immunofluorescence is highest in the hilar region. The box in (**i**) is magnified in (**j**–**m**). In the hilar region, Rogdi immunofluorescence surrounds MAP2-labelled dendrites and colocalises with the presynaptic marker Bassoon (**j**–**m**).

To test whether Rogdi is present at synapses in the brain, too, we immunostained sections of the hippocampus (Fig. 3). The stratum lucidum of the hippocampal CA3 region contains mossy fibre terminals, i.e. the unusually large excitatory presynaptic terminals of dentate gyrus granule cell axons, which contact the apical dendrites of CA3 pyramidal cells. Consistent with a localisation in mossy fibre terminals, Rogdi immunofluorescence was particularly prominent in the stratum lucidum (Fig. 3b, c). Higher magnification revealed that the Rogdi immunosignal colocalised with Bassoon and surrounded CA3 cell dendrites (Fig. 3d-g), indicating its enrichment at synapses. In the dentate gyrus, Rogdi appeared to be enriched in the hilus (Fig. 3h, i), where it again colocalised with Bassoon and surrounded dendrites (Fig. 3j-m). These data suggest that Rogdi is enriched at two prominent types of synapses in the hippocampus, i.e. mossy fibre and hilar synapses.

### Localisation of Recombinant GFP-Rogdi

**Figure 4:**
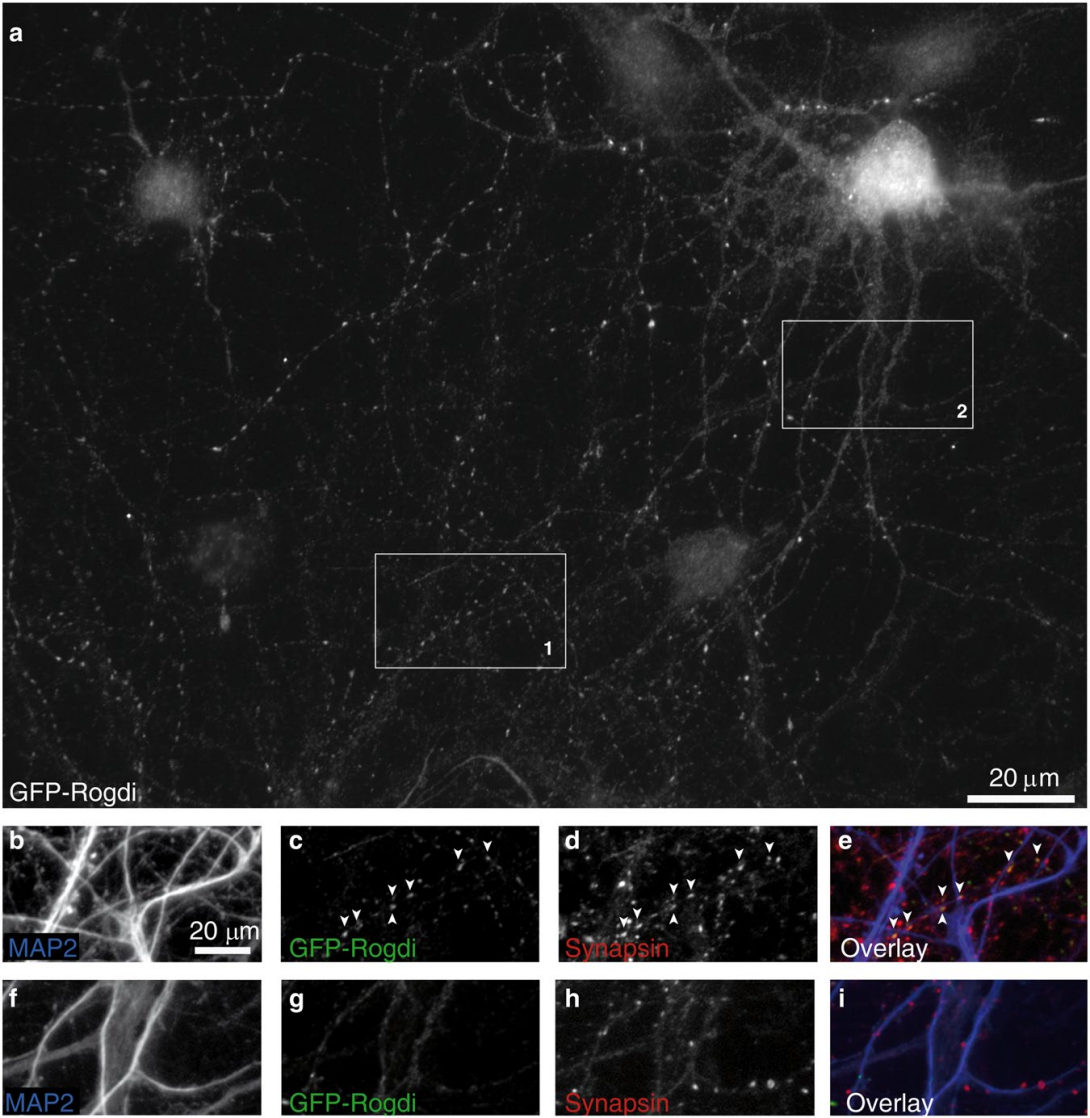
Presynaptic Targeting of GFP-Rogdi Expressed in Cultured Neurons. Localisation of GFP-Rogdi expressed in a cultured DIV 21 neuron. (**a**) Grey level image of GFP-Rogdi fluorescence in a transfected neuron. GFP-Rogdi fluorescence is punctate in areas distant from the soma. The distribution of these punctate signals in the field of view is characteristic of presynaptic localisation within a long and meandering process, i.e. the axon. GFP-Rogdi fluorescence is weaker and homogeneously distributed in the dendrites of the transfected neuron. The soma and nucleus are out of focus. (**b**–**e**) High magnification images of box 1. GFP-Rogdi puncta are located within a MAP2-negative process, colocalise with the synaptic vesicle protein Synapsin-1, and are in close proximity to MAP2-positive processes of non-transfected cells. (**f**–**i**) High magnification images of box 2. GFP-Rogdi fluorescence is diffusely distributed in dendrites of the transfected neuron, even where these dendrites are contacted by presynaptic vesicle clusters represented by Synapsin-1 puncta (for the original image see Fig. S6. For a comparison with another presynaptic GFP-tagged protein and with a postsynaptic GFP-tagged protein see Figs S7 and S8).

The colocalisation of Rogdi with synapse markers at the epifluorescence level may indicate pre- or postsynaptic localisation of Rogdi, or both. We previously used expression of GFP-tagged synaptic proteins in a small number of neurons to test whether a given recombinant protein is primarily targeted to presynaptic sites or to postsynaptic sites in any one neuron^11–13^. To test the targeting capacity of Rogdi, we expressed recombinant GFP-Rogdi in cultured neurons and determined its subcellular localisation. To this end, we used calcium phosphate transfection on DIV3. This transfection protocol yields sparse transfection and moderate expression levels, resulting in a minority of neurons being transfected, each with expression levels that do not saturate the endogenous targeting machinery^11,14^. In transfected DIV15 neurons, imaged at low magnification to observe the overall staining pattern, GFP-Rogdi was located in hot spots of fluorescence in a process that spanned more than 20 soma diameters and had a highly irregular course, i.e. the axon (Fig. 4a; see Fig. S6 for the original image of the entire field of view). Compared to this punctate fluorescence, weaker fluorescence was visible within the thicker processes originating from the soma, i.e. the dendrites (Fig. 4a). Higher magnification verified that punctate GFP-Rogdi fluorescence indeed occurred in MAP2-negative processes, i.e. axons, and colocalised with the synaptic vesicle protein Synapsin-1 at sites of contact with dendrites of non-transfected neurons (Fig. 4b-e). MAP2 staining also verified that the thicker processes that originated from the soma of a transfected neuron and contained weaker and diffuse fluorescence were indeed dendrites (Fig. 4f-i). Inside these dendrites, GFP-Rogdi fluorescence was diffuse even at sites where the dendrites received presynaptic inputs, identified by punctate Synapsin-1 staining (Fig. 4f-i). This indicates that the fraction of GFP-Rogdi found in dendrites does not accumulate at postsynaptic sites. In contrast, the axonally targeted fraction of GFP-Rogdi seems to accumulate at presynaptic sites.

The overall localisation pattern produced by GFP-Rogdi was strikingly similar to that of the presynaptic protein GFP-VAMP (Fig. S7), and clearly distinct from that of the postsynaptic protein PSD95-GFP (Fig. S8). Thus, both the distribution of GFP-Rogdi in transfected neurons and its co- accumulation with Synapsin-1 in axons, strongly suggest that GFP-Rogdi undergoes presynaptic targeting. This was further confirmed when we co-expressed GFP-Rogdi with Synaptophysin-mCherry, a red fluorescent variant of the synaptic vesicle protein Synaptophysin, known to target to presynaptic sites^15–17^; both proteins showed extensive colocalisation upon co-expression (Fig. S9). At DIV7, i.e. in immature neurons characterised by very short dendrites (approximately one soma diameter long), GFP-Rogdi produced punctate fluorescence in one long (more than 20 soma diameters long) process, presumably the axon (Fig. S10). Note that when using low magnification objectives, to capture both the cell body and the axonal arborisations of a neuron, the cell body is often out of focus and overexposed when using a focus and camera setting designed to detect the processes. When analysing the cell body selectively, we found that GFP-Rogdi was diffusely distributed in the soma and excluded from the nucleus (Fig. S10).

**Figure 5:**
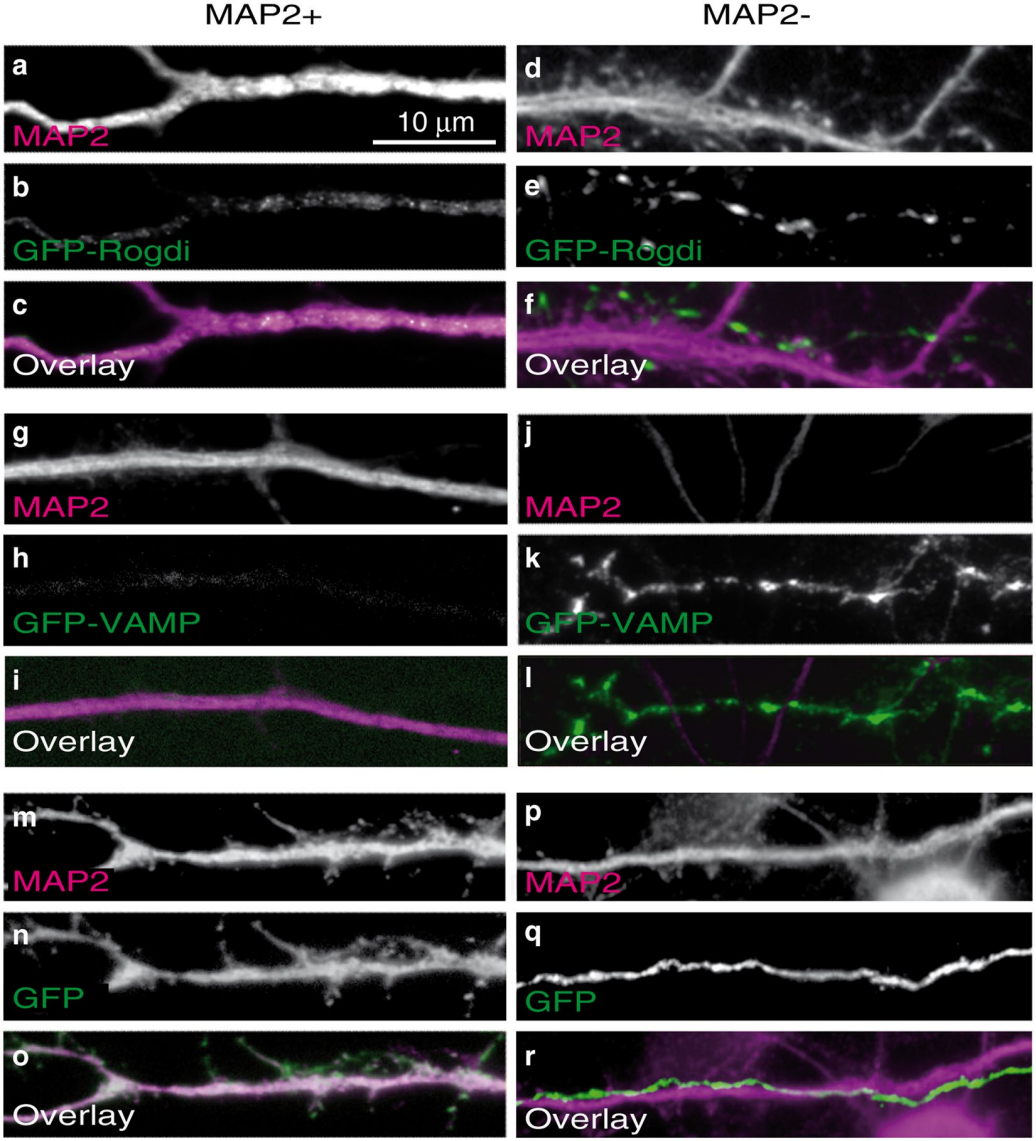
Distribution of GFP-Rogdi in Axons and Dendrites. Cultures were transfected with GFP-Rogdi (**a**–**f**), GFP-VAMP (**g**–**l**) or GFP alone (**m**–**r**), fixed, and immunostained. The left column shows representative examples of the distribution of these proteins in dendrites (MAP2 + processes), the right column shows representative examples of the distribution of these proteins in axons (MAP2- processes). The distribution of GFP-Rogdi resembled that of the synaptic vesicle marker GFP-VAMP, and differed from that of GFP. (**a**–**c**) Granular, homogeneously distributed GFP-Rogdi fluorescence in a dendrite. (**d**–**f**) Punctate GFP-Rogdi fluorescence in an axon running along a dendrite of an untransfected neuron. Note that MAP2 is primarily present in dendritic shafts. Spines are therefore weakly stained or not at all. Some presynaptic terminals may thus not be in direct apposition to MAP2 staining. (**g**–**l**) The distribution of the presynaptic protein GFP-VAMP resembled that of GFP-Rogdi (**h**–**i**) Granular, homogeneously distributed GFP-VAMP fluorescence in a dendrite. (**j**–**l**) Punctate GFP-fluorescence in an axon. (**m**–**o**) Homogeneous distribution of GFP in a dendrite. (**p**–**r**) Homogeneous distribution of GFP in an axon running along a dendrite of an untransfected neuron. The examples are representative of our observation that GFP-Rogdi was weak and difficult to distinguish from background in dendrites, but prominent and punctate in axons.

Having observed a distribution pattern of GFP-Rogdi consistent with presynaptic targeting, we analysed dendrites and axons of transfected cells at higher magnification (Fig. 5). These experiments revealed weak and somewhat granular GFP-Rogdi fluorescence homogeneously distributed inside dendrites (Fig. 5a-c). In contrast, axons were characterised by hotspots of locally increased fluorescence intensity (Fig. 5d-f). This staining pattern of GFP-Rogdi resembled that of GFP-VAMP, with weak fluorescence in dendrites (Fig. 5g-i) and intense puncta in axons (Fig. 5j-l). The staining pattern was clearly different compared to the diffuse distribution of GFP in dendrites (Fig. 5m-o) and axons (Fig. 5p-r). Moreover, it did not resemble the localisation of the postsynaptically targeted PSD95-GFP, which was absent from axons and punctate in the periphery of dendrites (Fig. S8). These data indicate that GFP-Rogdi is preferentially targeted to axons compared to dendrites. Within the axon, it appeared to be locally enriched in hot spots that may represent presynaptic specialisations.

**Figure 6:**
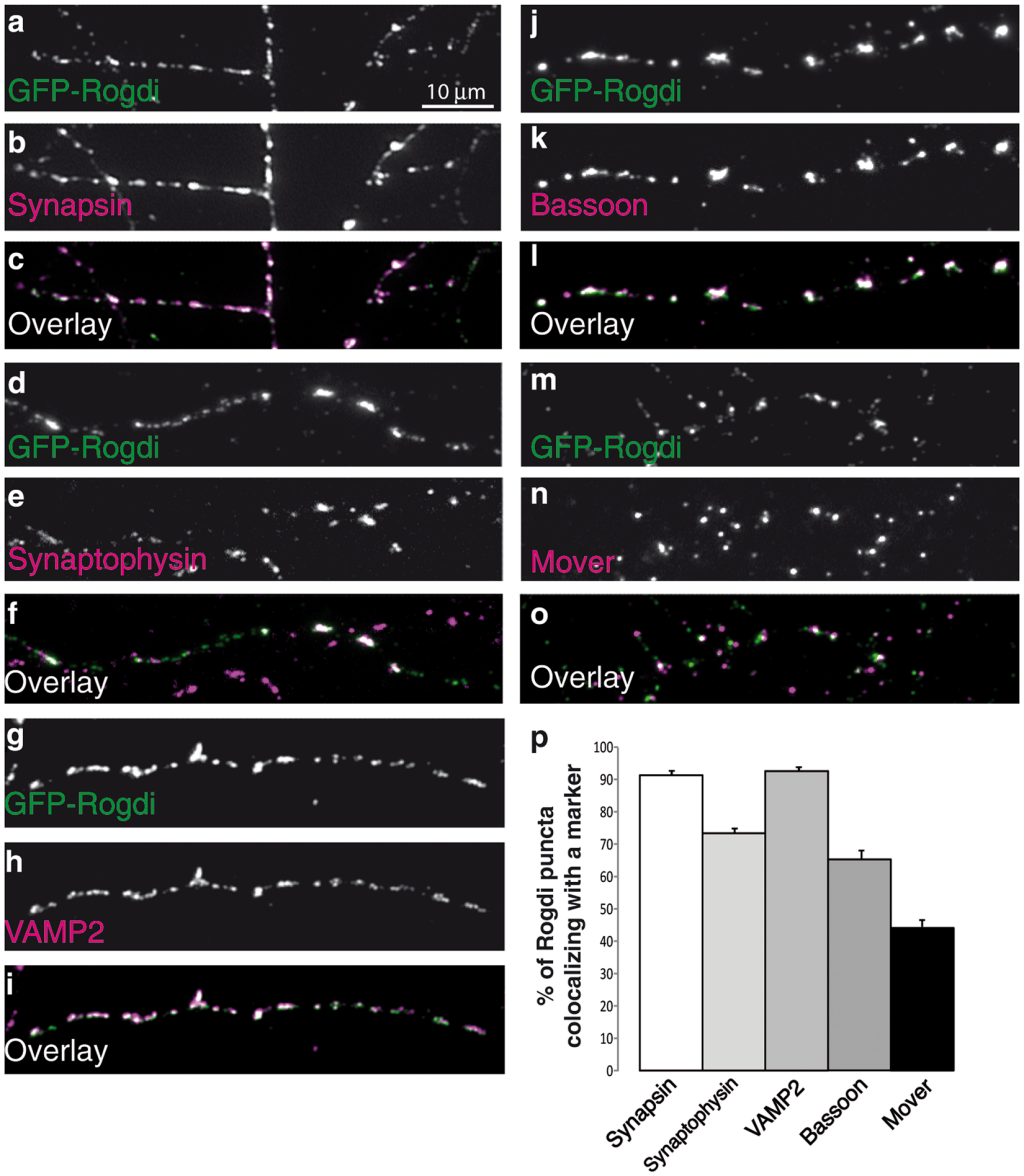
Axonal Puncta of GFP-Rogdi Colocalise with Synaptic Markers. GFP-Rogdi transfected cultures were fixed and immunostained for the markers indicated in the panels. (**p**) Quantification of the percentage of GFP-Rogdi puncta colocalising with the indicated markers. We performed 3 independent culture experiments (N = 3). In each experiment, 3 to 4 coverslips were analysed, adding up to 10 coverslips. On each coverslip, 3 areas were imaged, adding up to double-fluorescence images of 30 coverslip areas (n = 30). The average percent of colocalisation between GFP-Rogdi and a certain marker was determined. Error bars indicate SEM.

To test this notion, we immunostained GFP-Rogdi expressing cultures for endogenous markers of presynaptic terminals. We used Synapsin and Mover as examples of peripheral synaptic vesicle membrane proteins, Synaptophysin and VAMP2 as examples of synaptic vesicle transmembrane proteins, and Bassoon as an active zone scaffolding protein. GFP-Rogdi displayed extensive colocalisation with all presynaptic markers tested (Fig. 6). Note that the markers stain all synapses in the culture, including those in non-transfected cells. Immunosignals for markers that are not positive for GFP-Rogdi represent presynaptic terminals of untransfected cells. To estimate how many GFP-Rogdi puncta observed in the axon of any transfected neuron represent synapses, we determined the percentage of GFP-Rogdi puncta colocalising with a synapse marker (Fig. 6p). Colocalisation was particularly high with Synapsin, Synaptophysin and VAMP2 (91.24 ± 1.54% for Synapsin, 74.08 ± 2.04% for Synaptophysin, and 92.53 ± 1.35% for VAMP2), indicating that GFP-Rogdi accumulates at sites that contain synaptic vesicle clusters. Colocalisation was lower with Bassoon (66.26 ± 4.30%) and Mover (44.13 ± 4.04%).

**Figure 7:**
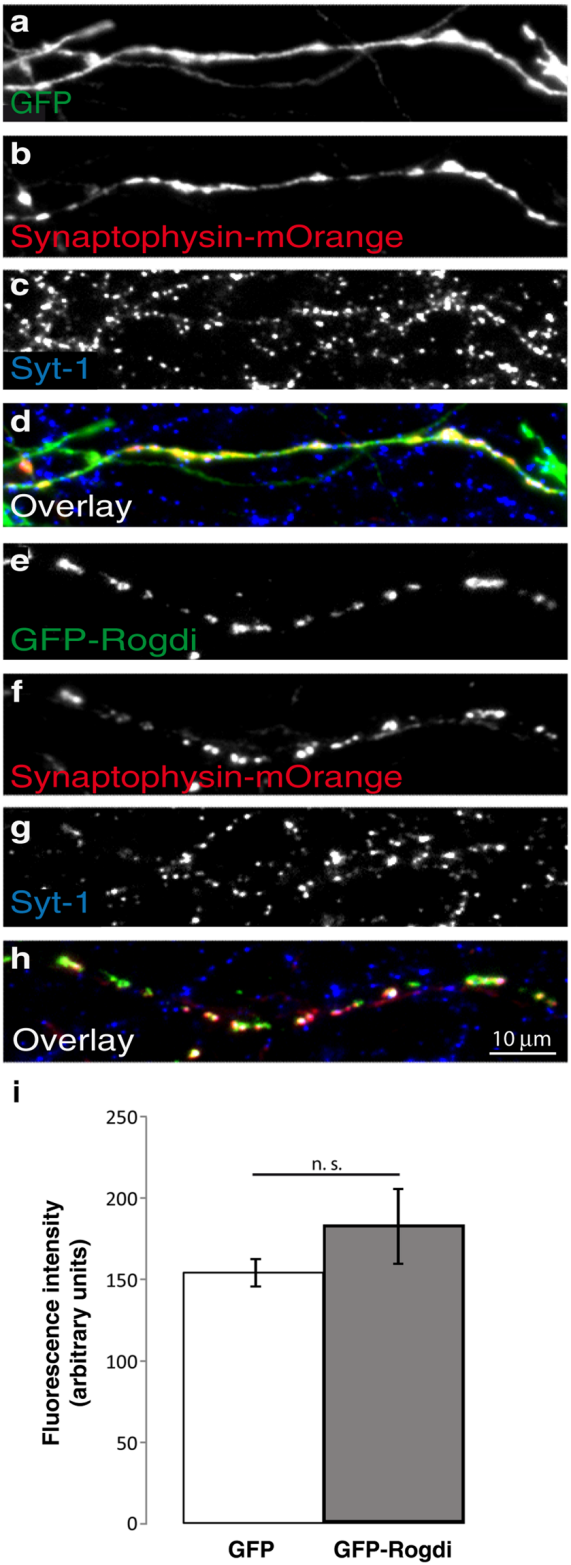
GFP-Rogdi is Targeted to Functional Synapses. Neuronal cultures were double transfected with either Synaptophysin-mOrange and GFP (**a**–**d**) or Synaptophysin-mOrange and GFP-Rogdi (**e**–**h**) and subjected to live Syt-1-uptake using a mouse monoclonal antibody directed against the luminal domain of Synaptotagmin 1. Cells were then fixed and immunostained using a rabbit anti-GFP antibody. Secondary antibodies were goat anti rabbit Alexa 488 to detect GFP and GFP-Rogdi, and goat anti mouse Alexa 647 to detect the Synaptotagmin 1 antibody taken up into synaptic vesicles during the live uptake assay. Synaptophysin-mOrange was detected based on mOrange autofluorescence. Synaptophysin-mOrange indicated the localisation of presynaptic sites, and GFP served as a control for the expression of GFP-Rogdi. Anti-Syt1 antibody was taken up by all active presynaptic sites following stimulation. Note that Syt1 uptake also occurs in axons of untransfected cells. As expected, GFP was diffusely distributed (**a**–**d**), while GFP-Rogdi was present at active presynaptic sites (**e**–**h**). (**i**) Average fluorescence intensity of Syt-1 antibody uptake at synapses in GFP expressing and in GFP-Rogdi expressing neurons. We performed 3 independent culture experiments (N = 3). In each experiment, 3 to 4 coverslips were analysed, adding up to 10 coverslips. On each coverslip, 3 areas were imaged, adding up to triple-fluorescence images of 30 coverslip areas (n = 30). Student’s *t*-test was performed. Error bars represent SEM, n.s. not significant.

To test whether GFP-Rogdi was targeted to functional synapses and whether it affected synaptic vesicle recycling, we employed a Syt1-uptake assay, i.e. an optical readout for depolarisation-dependent synaptic vesicle recycling (Fig. 7). The Syt1-uptake assay relies on the fact that antibodies directed against the luminal (intravesicular) domain of the synaptic vesicle transmembrane protein Synaptotagmin-1 are taken up into synaptic vesicles performing depolarisation-induced cycles of exo- and endocytosis in live cultures. During exocytosis, the luminal domain of Syt1 becomes exposed to the cell surface and captures anti-Syt1-antibodies applied to the culture medium. During endocytosis these antibodies are taken up into the presynaptic terminal, and can be detected by secondary antibodies after fixation. The intensity of the immunofluorescence signal for the Syt1-antibody is thus directly proportional to the extent of depolarisation-induced synaptic vesicle recycling. In these experiments, we co-expressed GFP or GFP-Rogdi with Synaptophysin-mOrange. In both GFP transfected and in GFP-Rogdi transfected neurons, Synaptophysin-mOrange indicated the localisation of synaptic vesicle clusters, and Syt-1-immunofluorescence indicated the extent of their recycling.

Quantification revealed that 57.63 ± 4.31% of GFP-Rogdi clusters colocalised with sites of Syt-1-uptake, indicating that more than half of the GFP-Rogdi puncta were localised to active synapses. Synaptophysin-mOrange positive synaptic vesicles clusters showed a similar extent of Syt-1-uptake, irrespective of whether GFP or GFP-Rogdi was co-transfected (Fig. 7i). The average fluorescence intensity generated by the internalised Syt1-antibody was 153.6 ± 8.8 arbitrary units in GFP-expressing cells, and 183.8 ± 23.4 arbitrary units in GFP-Rogdi expressing cells (p = 0.16; Student’s *t*-test; measurements are in arbitrary units). This indicates that moderate overexpression of GFP-Rogdi did not affect presynaptic terminal function. In addition, these experiments show that GFP-Rogdi is targeted to active presynaptic terminals.

## Discussion

The Rogdi gene is predicted to encode a 287 amino acid protein in human and rodents. Mutations in the gene cause Kohlschütter-Tönz syndrome^4,5^, but the underlying pathomechanisms have remained elusive. In particular, the properties of the corresponding Rogdi protein have been unknown. Here we report that Rogdi is a presynaptic protein.

Our conclusion is based on a combination of two approaches: first, using immunofluorescence, we detect punctate Rogdi immunosignals at sites positive for synaptic marker proteins in cultured neurons and in brain sections, indicating that Rogdi is present at synapses. Second, we used transfection of cultured hippocampal neurons and found that recombinant Rogdi accumulates at presynaptic but not at postsynaptic sites. Thus, Rogdi is endowed with the capacity for preferential targeting to presynaptic sites.

The punctate shape of the immunosignals for Rogdi – like that of the signals for the other markers, including Bassoon, Synapsin and PSD95 – indicates that these proteins are locally enriched at synapses. Interestingly, Rogdi immunosignals were homogeneously distributed inside axons and dendrites in young (DIV7) cultures, while they were punctate along the periphery of dendrites in older cultures. We cannot exclude that unspecific background contributes to the rather diffuse staining in young neurons. However, the shift from diffuse to punctate may represent redistribution from a ubiquitous localisation to a synaptic localisation during culture development. In any case, in mature neurons Rogdi was detected at synapses. Some of the synaptic Rogdi immunosignals colocalised with both Bassoon and the excitatory synapse marker PSD95, indicating that these Rogdi immunosignals were detected at excitatory synapses. Some Rogdi immunosignals colocalised with Bassoon only. Because Bassoon is a marker for both excitatory and inhibitory synapses, this is consistent with Rogdi being present at inhibitory synapses, too. Alternatively, these Rogdi-Bassoon double-positive sites may represent non-synaptic accumulations of the two proteins. Overall, our data indicate that in mature cultures, Rogdi immunosignals were found at excitatory synapses and may be present at inhibitory synapses, too. In sections from the hippocampus, Rogdi immunosignals were prominent in the stratum lucidum of the hippocampal CA3 region, where the unusually large presynaptic terminals of mossy fibres form excitatory contacts with CA3 pyramidal cells. This is also consistent with Rogdi being a component of excitatory nerve terminals.

An obvious caveat is the question whether our immunofluorescence analysis is specific for Rogdi. Several lines of evidence argue for the idea that the immunosignals indeed reflect the localisation of Rogdi: 1) Two distinct antisera yielded similar results, i.e. both yielded synaptic immunosignals in mature cultured neurons. The first antiserum was raised against an N-terminal region of human Rogdi. We tested it in our study and found that it detects recombinant rat Rogdi by western blotting and by immunofluorescence. The second antibody was raised against full-length human Rogdi and validated by knockdown of Rogdi previously^10^. In their study, Chen *et al*. used western blotting to show that reducing the levels of Rogdi by RNA interference reduced the band intensity for the Rogdi signal accordingly, arguing in favour of its specificity for Rogdi. 2) Both antibodies detected recombinant rat Rogdi by immunofluorescence in cultured neurons. Thus, both are suitable for immunofluorescence detection of Rogdi. 3) The antibody tested by us in this study detected a band of the expected size, i.e. 30 kDa, in western blots of protein lysates obtained from cultured hippocampal neurons and from rat brain synaptosomes, but no additional bands. The detection of a 30 kDa band in our cultured neurons and brain lysate is consistent with gene expression data suggesting that Rogdi is expressed in the brain, and that its expression levels are particularly high in the hippocampus^5,18^. We cannot exclude the possibility that the antibody cross-reacts with a protein other than Rogdi that has the same molecular weight and is synaptic. However, the ability of the antibody to detect recombinant Rogdi by western blotting and immunofluorescence and to detect a band of the expected size in western blots of nervous tissue renders it likely that the synaptic immunofluorescence signal actually represents the localisation of Rogdi.

To further address the caveat of antibody specificity, we employed a second approach to test whether Rogdi is a synaptic protein: we tested the targeting capacity of recombinant Rogdi and found that it accumulates at presynaptic sites. Two aspects of this observation are worth mentioning: first, it further corroborates the data obtained by immunofluorescence of endogenous Rogdi. Second, it adds strong evidence for Rogdi being a presynaptic rather than a postsynaptic protein. We had previously used this approach to analyse the presynaptic targeting behaviour of recombinant versions of the active zone scaffolding protein Bassoon^11,14,19^, the synaptic vesicle proteins Mover and VAMP2^12,20,21^, presynaptic transmembrane proteins from the Neurexin family^13^, as well as the postsynaptic targeting of Neuroligin-1, PSD95 and ProSAP1^22–24^. In all of these cases, the tags did not interfere with the targeting of the proteins. This is also obvious from the correct targeting behaviour of GFP-VAMP, PSD95-GFP, Synaptophysin-mOrange and Synaptophysin-mCherry in our present study (see references for further examples in the supplemental data). It appears unlikely that in the case of Rogdi the GFP tag has dramatic effects, in that it both abolishes postsynaptic targeting and creates presynaptic targeting. We cannot exclude, however, that the GFP tag is permissive for Rogdi’s presynaptic targeting capacity, but prevents postsynaptic targeting. If this was the case, Rogdi might be present at both pre- and postsynaptic sites.

The fact that GFP-Rogdi showed a presynaptic localisation pattern, colocalised with synaptic markers in axons, and accumulated at axonal sites of active synaptic vesicle recycling underscores that Rogdi is endowed with presynaptic targeting signals. This is further supported by the observation that GFP-Rogdi co-distributed with established reporters of presynaptic accumulation, i.e. Synaptophysin-mCherry and Synaptophysin-mOrange^15–17,25^ in co-expression experiments. Presynaptic targeting of Rogdi is consistent with the detection of Rogdi by mass spectrometry in synaptic vesicle fractions from rat brain^8^. Our data extend these mass spectrometry data by indicating that Rogdi is not only present at presynaptic terminals, but in fact contains targeting capacity for presynaptic accumulation.

Recombinant Rogdi was reported to accumulate in the nucleus of HEK293 cells, and endogenous Rogdi was reported to colocalise with the nuclear envelope marker LAMIN A in blood mononuclear cells and skin fibroblasts by immunofluorescence^5^. We did not observe evidence for a nuclear localisation of the endogenous Rogdi or GFP-Rogdi. However, we cannot exclude that Rogdi has several conformations and that the antibodies used here selectively detect presynaptic Rogdi. This scenario appears unlikely, however, as the two antisera were raised against different epitopes, and one of the antisera was raised against the full length protein. Alternatively, Rogdi may undergo neuron-specific subcellular trafficking, i.e. targeting to presynaptic sites may override nuclear targeting in neurons. In addition, Rogdi may shuttle between presynaptic terminals and the nucleus, depending on the activity-status of the neuronal network, as has previously been shown for the protein CtBP1^26^. A presynaptic localisation of Rogdi is consistent with its yeast-2-hybrid interaction with DISC1^27^, a presynaptic scaffolding protein^28–30^, that could thus anchor Rogdi in presynaptic terminals.

As a presynaptic protein, Rogdi is unlikely to be a gene regulatory protein (but such a role is not excluded: see above). If Rogdi does not control a large number of genes, how do mutations in Rogdi cause the complex aspects of Kohlschütter-Tönz syndrome, including amelogenesis imperfecta, epilepsy, and psychomotor delay? One possibility is that Rogdi may regulate exocytosis in neurons and ameloblasts. Exocytosis of neurotransmitter and synaptic vesicle recycling are hallmarks of presynaptic function at mature synapses. In addition, axons use exocytosis to secrete neurotrophins and synaptic cleft material, and to incorporate presynaptic cell adhesion molecules in the plasma membrane during synapse formation. Moreover, before synapse formation, axons release guidance cues and extracellular matrix proteases during pathfinding. A role for Rogdi in one or more of these exocytotic events is an attractive working hypothesis, as it may unify the roles of Rogdi in presynaptic nerve endings and in ameloblasts through a common mechanism, i.e. exocytosis. In their secretory phase, ameloblasts secrete matrix metalloproteinases to promote enamel formation^31^, which is impaired in Kohlschütter-Tönz syndrome, leading to the clinical manifestation of amelogenesis imperfecta. Matrix metalloproteinases are also regulators of neuronal development^32^. In addition, ameloblasts express N-Cadherin, a transmembrane cell adhesion molecule crucial for brain development and synapse function^33^. It appears plausible to assume that dysfunctional exocytosis of any of these compounds would affect enamel deposition, neural development and synaptic function.

Why does the loss of one presynaptic protein lead to such devastating neurological symptoms as the ones observed in Kohlschütter-Tönz syndrome? Epilepsy has been observed in mice mutants for the presynaptic scaffolding protein Bassoon^34^. It would be interesting to test whether Rogdi is expressed in a subset of synapses, e.g. at inhibitory terminals, where its loss could cause an imbalance between excitation and inhibition. In addition, by interfering with exocytotic events, Rogdi could affect neuronal migration, axonal pathfinding or synapse formation in a subset of neurons early during development, causing a neurodevelopmental phenotype. Knockout studies should reveal the temporal and spatial aspects of its role, as well as the underlying molecular and cellular mechanism.

Overall, our data introduce Rogdi as a novel component of presynaptic nerve terminals, and add a new perspective to unravelling the pathomechanisms underlying Kohlschütter-Tönz syndrome.

## Materials And Methods

### Study Approval

No studies with live animals were conducted. Experiments involving killing of animals to obtain brain samples and cell cultures were approved by the local animal protection authorities (Tierschutzkommission der Universitätsmedizin Göttingen) under the approval number T 10/30. The experiments were conducted in accordance with the approved protocols.

### Plasmids

All recombinant constructs were cloned into the pXFP (Clontech) vector backbone and driven by the CMV promotor. In the paper, we refer to all variants of EGFP used as GFP. The monomeric variant of EGFP (EGFP A207K mutant) was fused to the N-terminus of full length rat Rogdi. Full length rat Rogdi was produced by gene synthesis (Genscript, USA). As a control, we used the monomeric variant of EGFP alone. Conventional EGFP was fused to the N-terminus of a PCR product encoding rat VAMP-2/Synaptobrevin-2. PSD95-EGFP is described in^23^. Synaptophysin was tagged with mCherry or mOrange at its C-terminus. Synaptophysin-mOrange is a kind gift of L. Reichardt^25^.

### Antibodies

We used the following mouse monoclonal antibodies: anti-Bassoon (Enzo, Life Sciences cat. no.: O88778); anti-Synapsin, anti-Synaptophysin, anti-Synaptotagmin-1 (Synaptic Systems cat. no.: 106001, 101011, 104202); anti-TGN38 (BD Biosciences cat. no.: 610899). In addition we used the following rabbit polyclonal antibodies: anti-GFP 6556 (Abcam cat. no.: ab6556); anti-MAP2, anti-Mover, anti-TRIM 36, anti-VAMP2, (Synaptic Systems cat. no.: 188004, 248003, 377003, 104203); anti-Rogdi, directed against amino acids 14–95 of human Rogdi (Sigma-Aldrich cat. no.: hpa041000); anti-Rogdi, directed against full-length human Rogdi GST-fusion protein (Proteintech cat. No.: 17047–1-AP), validated by knockdown^10^. In addition, we used the following guinea pig polyclonal antibodies: anti-MAP2, anti-Neurofilament H (Synaptic Systems cat. no.: 188004, 171104).

### Biochemistry

To obtain total protein from cultured neurons, cultures were washed with PBS and incubated with 80 µl per well 1x SDS sample buffer containing Tris, glycerin, SDS, bromophenol blue, and ß-mercaptoethanol for 5 min. The solution was transferred from one well to the next every 5 min to receive a sufficient concentration of protein. The sample was heated at 95 °C for 5 min and centrifuged at 1000 g for another 5 min afterwards. The supernatant was subjected to SDS-PAGE, western blotting and ECL detection. Synaptosomal fractions from the brains of adult rats were obtained from Synaptic Systems (Göttingen, Germany. Cat. No. 502-P2).

### Cell Culture

Primary cultured hippocampal rat neurons were obtained from embryonic day 19 rat brains as described in Dresbach *et al*.^11^. Briefly, after dissection, hippocampi were incubated with 0.25% trypsin for 20 min at 37 °C. Samples were then washed three times using HBSS to remove trypsin. For mechanical dissociation the samples were triturated using a syringe and two different sized needles for three times each. The solution was passed through a strainer and stored in a 50-ml-Eppendorf tube. Neurons were plated onto PEI (poly ethylene imine) coated coverslips at a density of 50,000 cells per cover slip in 24-well-plates. For storage the plates were incubated at 37 °C, 95% humidity and 5% CO_2_ in culture medium containing Neurobasal® medium, 2% B27, 1% Penicillin-Streptomycin and 0.25% L-glutamine.

HEK293-cells were maintained in DMEM, 10% fetal calf serum, 1% Penicillin-Streptomycin and 0.25% L-glutamine. The reagents and media were purchase from Gibco/Invitrogen (HBSS, Neurobasal®, OptiMEM®, B27, Penicillin-Streptomycin, DMEM) and Biochrome (fetal calf serum).

### Transfection

#### Calcium phosphate transfection of neurons

To obtain low amounts of expression in a small number of neurons, cultures were transfected using calcium phosphate precipitation on DIV3^9^. Briefly, the culture medium was exchanged for 500 µl OptiMEM® pre-warmed to 37 °C. Neurons were kept in OptiMEM® in the incubator for 30 to 60 minutes. The original medium was stored in the incubator. To prepare the precipitate for 7 wells, 105 µl of transfection buffer (274 mM NaCl, 10 mM KCl, 1.4 mM Na_2_HPO_4_, 15 mM Glucose, 42 mM HEPES, pH 7.06) were added dropwise to 105 µl of a solution containing 7 µg of DNA and 250 mM CaCl_2_, under gentle stirring. For co-transfections, 7 µg of each DNA were used. The resulting mix was placed for 20 minutes at room temperature in the dark. 30 µl of the mix (including 1 µg DNA per construct) were then added per well, and neurons were placed in the incubator for 60 to 90 minutes. Medium was then gently exchanged for 1 ml 37 °C pre-warmed Neurobasal®, followed by two 750 µl exchanges. Finally, Neurobasal® was exchanged for the stored culture medium, and the neurons were stored in the incubator until used. In our hands, this protocol yields a relatively low level of expression in each transfected cell and a relatively small number of transfected cells per coverslip. On average 0.1% of the neurons (including excitatory and inhibitory neurons) become transfected. The cultures contain excitatory and inhibitory neurons. This prevents overtaxing of the cellular transport mechanisms, and allows for the tracking of processes of individual transfected cells^11,14,21^.

#### Lipofectamine 2000® transfection of neurons

To obtain high amounts of overexpression in cultured neurons, we used Lipofectamine 2000® (ThermoFischer Scientific), according to the manufacturers protocol. Lipofectamine 2000® transfections of cultured neurons were performed on DIV6, and the neurons were fixed and analysed DIV7. Briefly, culture medium was replaced with 1 ml fresh, pre-warmed culture medium without antibiotics, and both the neurons and the removed culture medium were stored in the incubator for 1 hour. Transfection mixes were prepared by adding 75 µl OptiMEM® and 3 µl Lipofectamine 2000 to one reaction tube, and 75 µl OptiMEM® and 3 µg DNA to a second reaction tube. The contents of the two tubes were mixed and stored for 20 minutes at room temperature. 50 µl of the transfection mix were then added per well, and the neurons were placed in the incubator for 90 minutes. The neurons were washed three times with Neurobasal®. The original medium was then added back to the wells, and the neurons were kept in the incubator for 24 hours. In our hands, this protocol yields high enough levels of overexpression in each transfected cell to overtax the endogenous targeting machinery and cause the neurons to be filled with recombinant protein throughout. Note that Lipofectamine 2000® can be used to generate moderate expression levels using different protocols.

#### PEI-transfection of HEK293-cells

HEK293-cells were transfected by mixing 15 µl OptiMEM®, 1 µg DNA and polyethylenimine (PEI) to yield a total volume of 30 µl.

### Immunofluorescence and Fluorescence Imaging

*Cultures* For fixation, neurons were treated with 4 °C -cold 4% paraformaldehyde (PFA) for 20 min. For triple stainings including PSD95, a 15 minute methanol incubation step was added after PFA fixation. After washing (3 × 5 min in PBS) and incubation with blocking solution, including fetal bovine serum, sucrose, BSA and tritonX-100 in PBS for 30 min, primary antibodies were applied in blocking solution over night at 4 °C. GFP and GFP-tagged proteins were visualised using anti-GFP immunofluorescence. Secondary antibodies conjugated to either Alexa 488, Cy3 or Alexa 647, were applied for 60 min at RT. Afterwards, Mowiol was used for mounting the coverslips. Cells were viewed by using 10x, 40x oil and 63x oil immersion objectives, and pictures were taken with a PhotometricsCoolSNAP HQ2 (Photometrics) in a Zeiss AxioObserver Z1microscope (Software VisiView).

*Brain sections* Adult Wistar rats were perfused transcardially with 4% PFA in PBS for 20 min. Brains were isolated and postfixated in 4% PFA for 24 hours at 4 °C. Vibratome sections (75 µm) were pre-blocked for 90 min and incubated with relevant primary antibodies overnight at 4 °C. After washing, sections were incubated with secondary antibodies, counterstained with DAPI, mounted with Mowiol + DABCO and analysed with epifluorescence.

### Syt-1-Uptake

For testing synaptic activity we performed a Synaptotagmin-1-uptake (Syt1-uptake) assay^9^. Synaptic terminals were depolarised for 5 minutes in the incubator by using a stimulation buffer containing 64 mM NaCl, 70 mM KCl, 1 mM MgCl_2_, 2 mM CaCl_2_, 20 mM HEPES and 30 mM glucose, and anti-Synatptotagmin antibody (Synaptic Systems) diluted at 1:600. After washing three times with Neurobasal® medium cells were fixed and processed for immunostaining. The internalised anti-Synaptotagmin antibody was detected using a secondary antibody labelled with Alexa 647.

### Statistical Analysis

Quantification and statistics were done with OpenView (written by Noam E. Ziv, Technion – Israel Institute of Technology, Haifa, Israel^35^), Microsoft Excel and IBM SPSS statistics 21. For each analysis, we performed 3 independent culture experiments (N = 3). In each experiment, 3 to 4 coverslips were analysed, adding up to 10 coverslips. On each coverslip, 3 areas were imaged, adding up to double- or triple-fluorescence images of 30 coverslip areas (n = 30).

To assess the colocalisation of GFP-Rogdi puncta with synaptic markers, we designed the following work flow: We recorded images in two fluorescence channels for each area. The first channel represented GFP-Rogdi fluorescence, the second channel represented the fluorescence of one marker. The subsequent steps were performed using OpenView software: For one area of interest showing GFP-Rogdi, we set the lower-intensity threshold such that diffuse fluorescence was excluded, leaving only punctate signals in the image. Next, we checked whether this threshold yielded a similar inclusion of punctate signals and exclusion of diffuse fluorescence in other areas on the same and on different coverslips. Once an appropriate threshold was identified, this threshold was applied to all 30 GFP-Rogdi images, producing punctate regions of interest (ROIs) in the GFP-Rogdi channel. Next, we transferred these ROIs to the corresponding channel 2 of each coverslip area, to determine whether there was marker fluorescence in the same ROIs or not. For each ROI, we logged the average channel 2 fluorescence intensity. In addition, we placed three ROIs in channel 2 at sites of the image that obviously showed only background fluorescence (i.e. no cells, no processes and no puncta) and logged these, too. We calculated the average background for channel 2 and subtracted this value from every ROI. The remaining value indicated whether any ROI had fluorescence in channel 2 that was above background or not. To make this stringent, we decided that the fluorescence of an ROI had to be at least 33% higher than background to count as positive. The percentage of positive ROIs of the total number of ROIs was then calculated. This represented the percentage of GFP-Rogdi puncta colocalised with a given marker.

To determine the colocalisation of GFP-Rogdi with sites of Syt1-uptake, we applied the same workflow. Channel 1 contained the GFP-Rogdi signal, channel 2 contained the Syt1-uptake immunosignal.

To determine the extent of Syt1-uptake in axons of cells expressing either GFP or GFP-Rogdi, we used three channels. Channel 1 represented green fluorescence (generated by GFP immunofluorescence), channel 2 represented Synaptophysin-mOrange autofluorescence, channel 3 represented the Syt1-uptake immunosignal (Alexa 647-fluorescence). In this case, we used Synaptophysin-mOrange to threshold the images, i.e. to generate ROIs at synaptic sites. For every ROI generated, we verified that the Synaptophysin-mOrange signal was in a double-transfected cell, by testing whether the corresponding channel 1 signal (GFP immunofluorescence) was above background. We transferred these ROIs to the Syt1-uptake channel and determined the average fluorescence intensity in each ROI. We then compared the average fluorescence intensity of the Syt1-uptake immunosignals of cells expressing GFP to that of cells expressing GFP-Rogdi.

For all colocalisation studies we performed a χ^2^-test to determine statistical significance. The two conditions for the χ^2^-test were defined as 1 = colocalisation and 0 = no colocalisation. The examination of the results from the Syt-1-uptake was done using the Student’s *t*-test.

## Electronic supplementary material

Supplementary Data
