# Supplementary material for: The Kohlschütter-Tönz syndrome associated gene Rogdi encodes a novel presynaptic protein
Source: Sci Rep. 2017 Nov 17;7:15791. doi: 10.1038/s41598-017-16004-1 (PMC5693994; doi:10.1038/s41598-017-16004-1)
Supplement: Supplementary file 1 — Supplementary Data [file 41598_2017_16004_MOESM1_ESM.pdf]

The Kohlschütter-Tönz syndrome associated gene Rogdi encodes a novel presynaptic protein

Donatus Riemann, Rebecca Wallrafen, Thomas Dresbach

Supplementary Information

Supplemental data

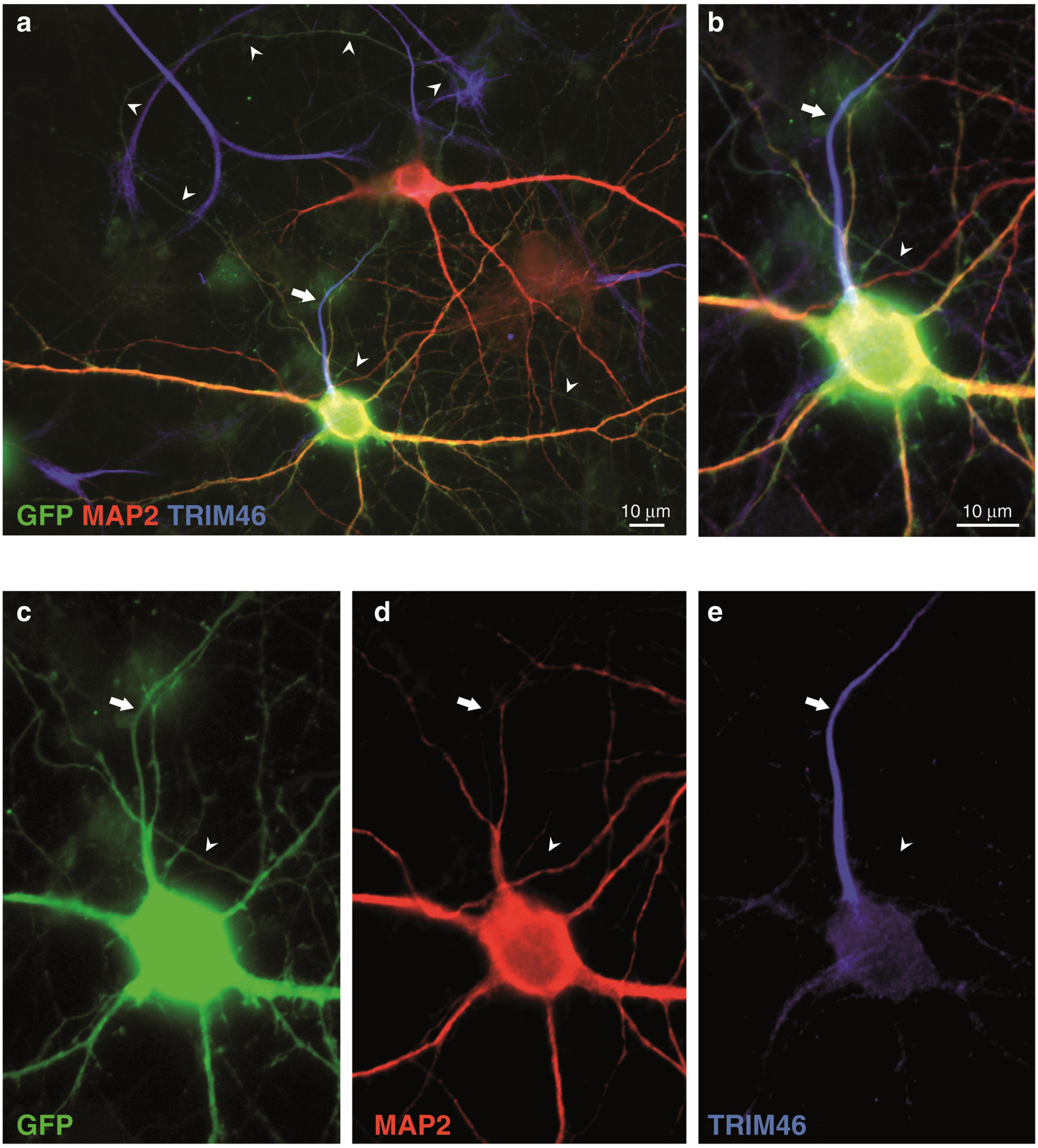

Figure S1:

Validation of Compartment Markers, Part 1: MAP2-Negative Processes are Axons

(a) Triple fluorescence of a neuronal culture transfected with GFP using the calcium-phosphate method. GFP was used to fill and detect all processes of a transfected neuron. MAP2 labels dendrites, the soma, and a short, proximal part of the axon. TRIM46 labels proximal parts of the axon. (a) The neuron in the bottom centre expresses GFP. The MAP2-

labelled dendrites of the transfected cell are seen in yellow / orange (resulting from red MAP2 and green GFP). The blue TRIM46 immunosignal indicates where the axon originates from its soma. The arrow indicates a region where TRIM46 is present in the proximal axon. The arrowheads indicate the course of the more distal parts of the axon, where TRIM46 is absent: the axon of the transfected neuron runs upwards in the panel, then to the left and then turns right to pass the soma of the transfected cell. The axon is the MAP2-negative process of the transfected neuron. (b-e) Magnified images.

Supplemental Information to Figure S1:

MAP2 staining is a commonly used means to distinguish dendrites, i.e. MAP2-positive processes, from axons , i.e. MAP2-negative processes (see e.g. [1–4]). The rationale is that the axons of the more than 50.000 neurons on a 12mm diameter coverslip form an increasingly dense neuropil during culture development. Staining axons directly using an axon marker usually makes it impossible to prove that a certain process containing a GFP-tagged protein is an axon, because dozens to hundreds of axons from untransfected neurons run in parallel with the transfected axon, often crossing it. Thus, it is not clear whether the axon marker is in the transfected process or just next to it or crossing it. This problem is particularly paramount in advanced culture stages that are needed to test for the presence of synapses.

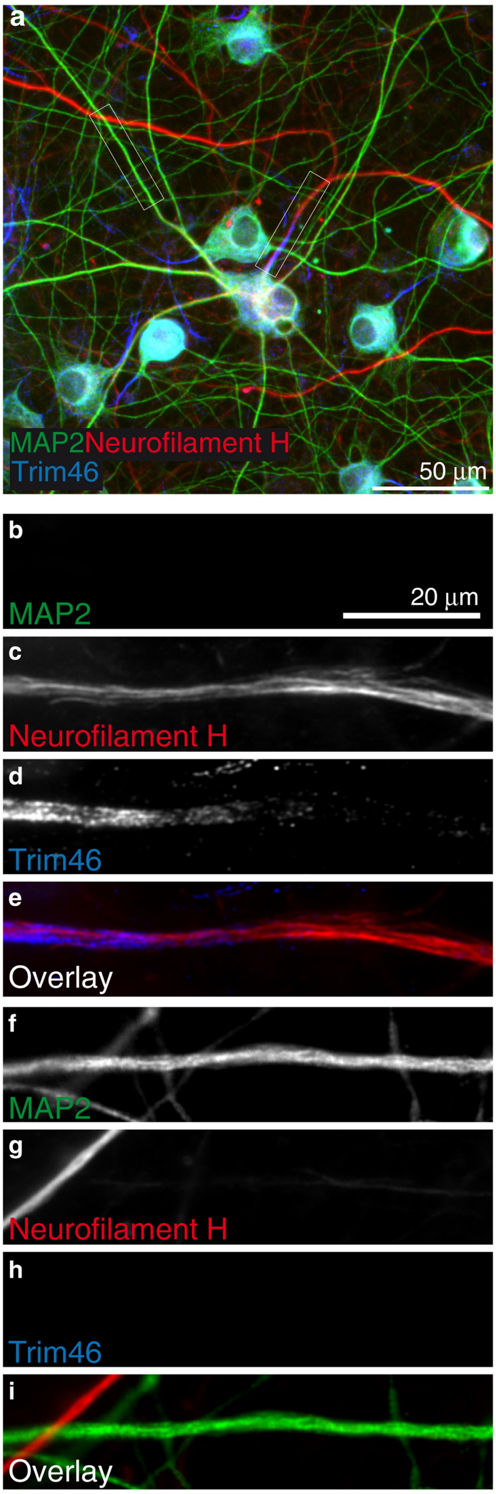

Figure S2:

Validation of Compartment Markers, Part 2: MAP2-Negative Processes are Axons

(a) Triple fluorescence of an untransfected neuronal culture. MAP2 labels dendrites, the soma, and a short, proximal part of the axon. TRIM46 labels proximal parts of the axon. Neurofilament H is used to label axons. (a) Overview. The right box is magnified in b-e, the

left box is magnified in f-i. (b-e) TRIM46 indicates the proximal part of the axon. Neurofilament H is present in the axon, MAP2 is absent. Thus, MAP2-negative processes are axons. (f-i) MAP2-positive processes are negative for TRIM46 and Neurofilament H. This experiment verifies that MAP2 staining suffices to distinguish dendrites from axons.

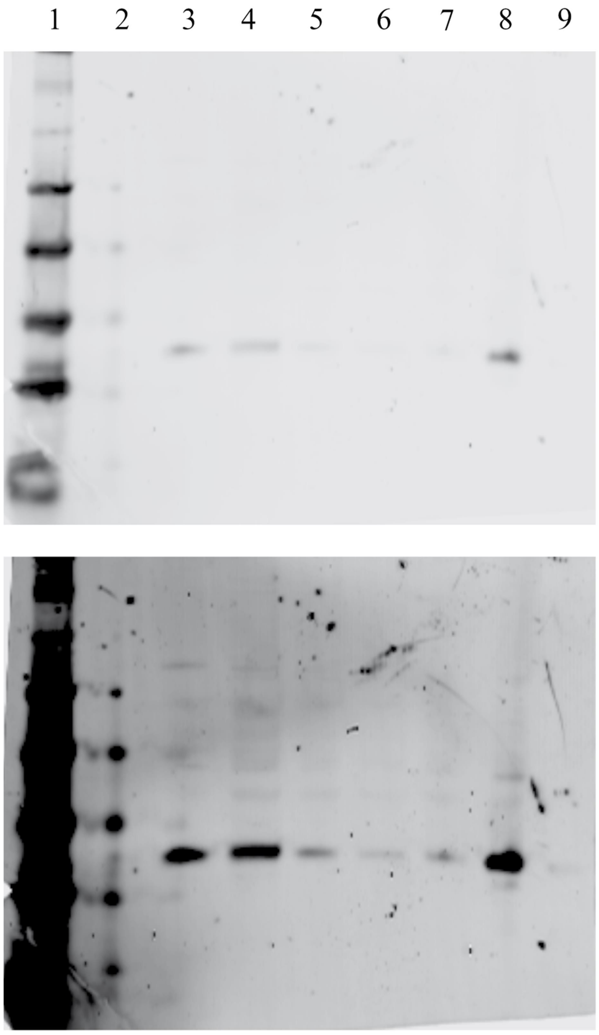

Figure S3:

Two Exposure Times of the Original of the Cropped Western Blot Shown in Figure 1p

Figure 1p shows lanes 1,4 and 8 of this blot.

Lane 1: SeeBlue Pre-Stained marker (Novex Life Technologies; cat. no. LC 5625)

Lane 2: no sample applied

Lane 3: hippocampal culture lysate 1

Lane 4: hippocampal culture lysate 2

Lane 5: hippocampal culture lysate 2 diluted

Lane 6: cortical culture lysate 1

Lane 7: cortical culture lysate 2

Lane 8: rat brain synaptosomes

Lane 9: no sample applied

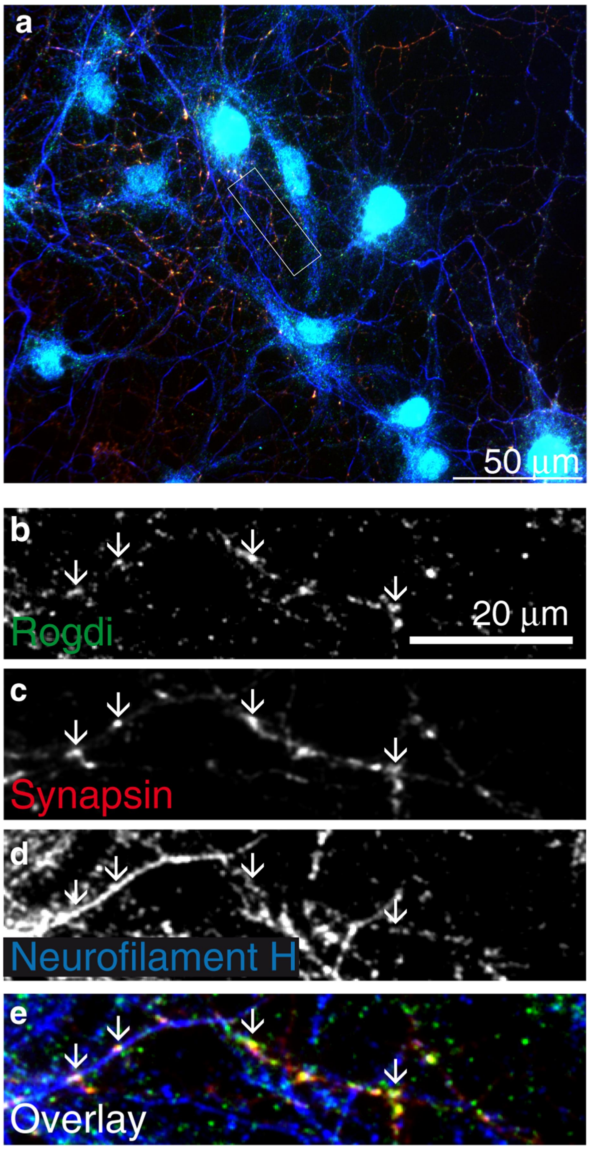

Figure S4:

Rogdi Immunosignals are Detected in Neurofilament H Positive Processes

(a) Triple fluorescence of an untransfected neuronal culture. Neurofilament H is used to label axons. Synapsin is a synaptic vesicle protein used to detect synapses. (a) Overview. The cell bodies are out of focus. (b,c) Examples of colocalisation of punctate Rogdi immunosignals with Synapsin are indicated by arrows. (d,e) Colocalisation between Rogdi and Synapsin occurs in Neurofilament H positive structures, i.e. axons.

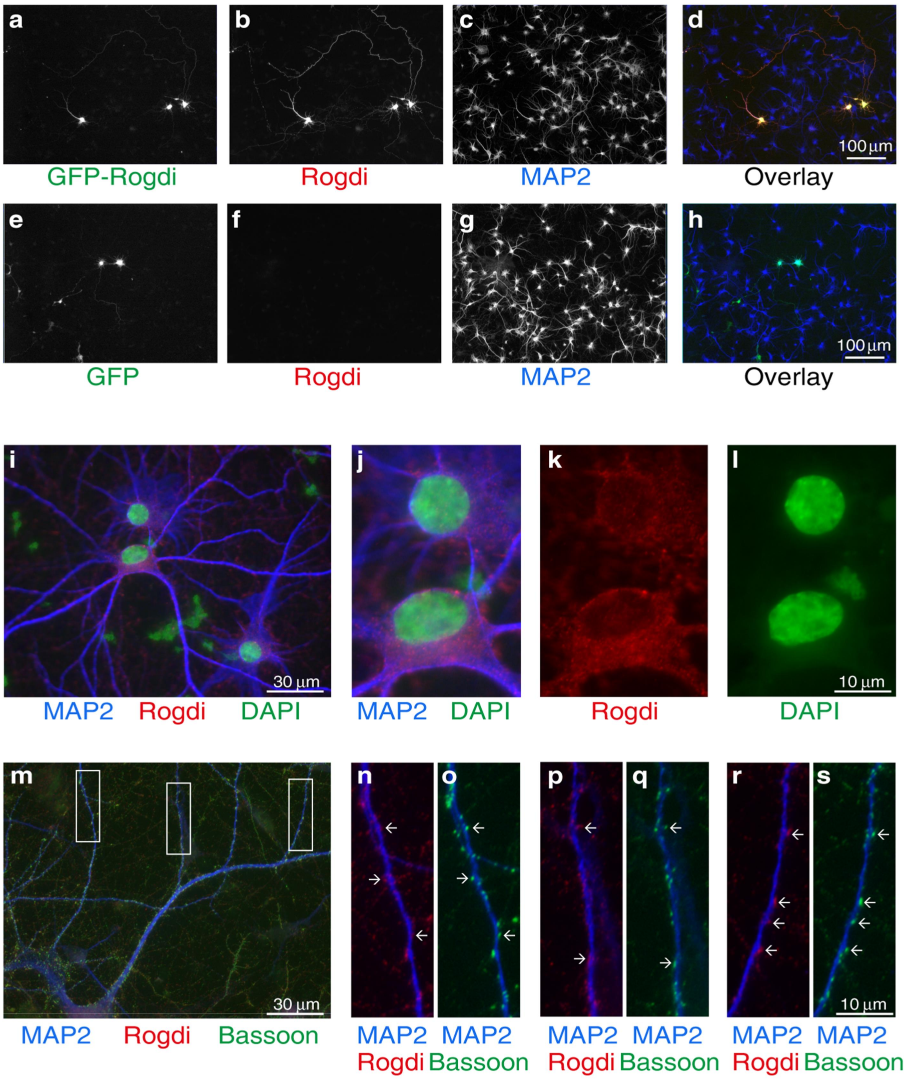

Figure S5:

A Knockdown-Validated Antibody Raised against Full-Length Human Rogdi Detects Recombinant Rat Rogdi and Produces Synaptic Immunosignals in Untransfected Cultures

(a-h) Triple fluorescence of cultures transfected with GFP-tagged rat Rogdi or GFP using the Lipofectamine 2000 method. MAP2 staining indicates all neurons in the field of view. The knockdown-validated antibody [5] (purchased from Proteintech) detects GFP-Rogdi, but not GFP. (i-l) The antibody produces a homogeneously distributed, granular staining in the soma of untransfected DIV15 neurons, and appears to be absent from the nucleus. DAPI is used to detect nuclei. Note that in panels (i-l) the soma and nucleus are in focus, while dendritic and potentially synaptic areas are out of focus. (m-s) In these panels, dendrites and potentially

synaptic areas are in focus. The left box in (m) is magnified in (n,o), the middle box is magnified in (p,q), the right box is magnified in (r,s). Arrows indicate examples of colocalisation between punctate immunosignals for Rogdi and Bassoon at dendrites.

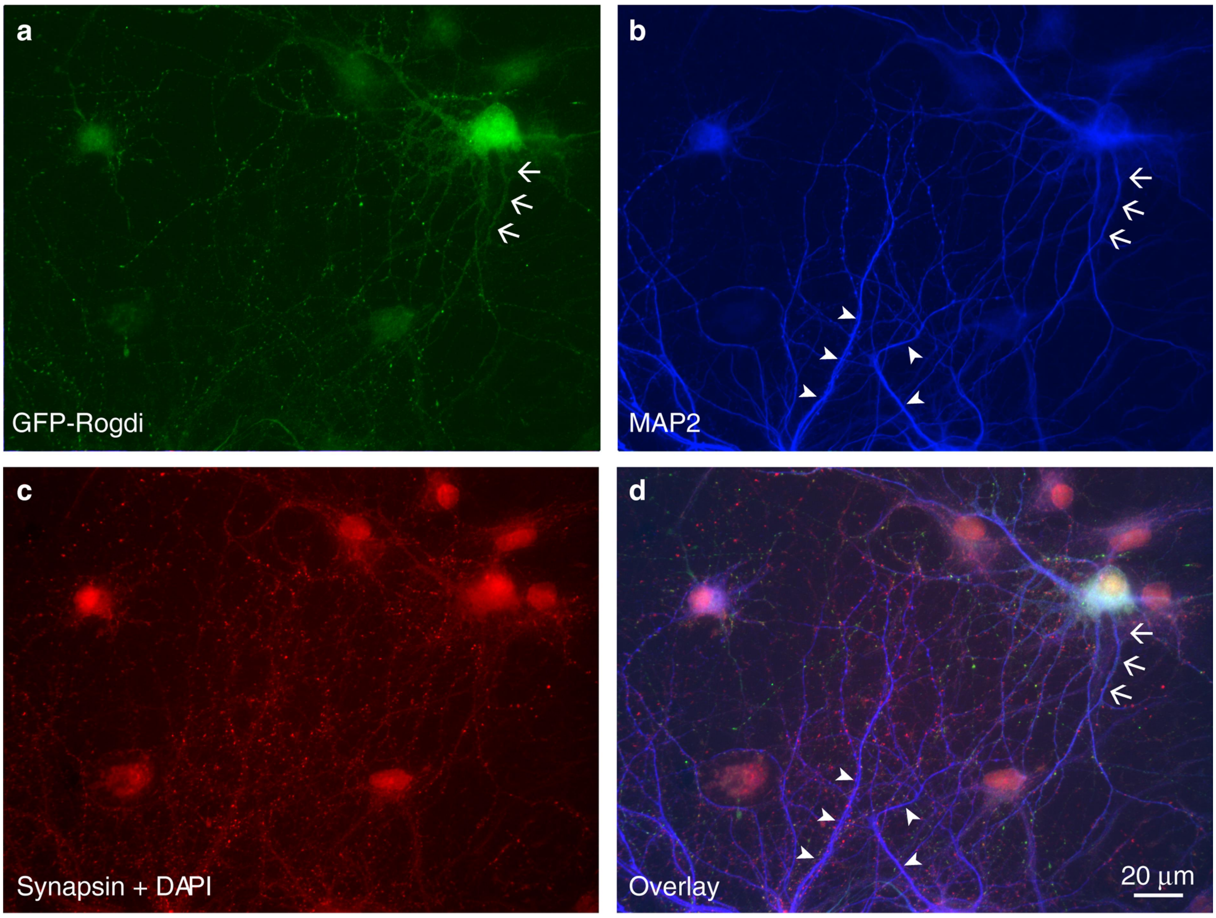

Figure S6:

Original of the Images Displayed in Figure 4

Quadruple fluorescence of a neuron from a DIV21 culture transfected with GFP-Rogdi using the calcium-phosphate method. MAP2 indicates all dendrites in the field of view, including those of the transfected neuron. DAPI was used to detect nuclei. We gave the DAPI signal (originally detected in the UV channel) red pseudocolour to avoid having four different colours in the overlay image (d). A grey level image of (a) is shown in Figure 4a. Arrows point at a dendrite of the transfected neuron. This dendrite is included in box 2 of Figure 4a. Arrowheads point at dendrites coming from untransfected neurons outside the field of view. This area is included in box 1 of Figure 4a.

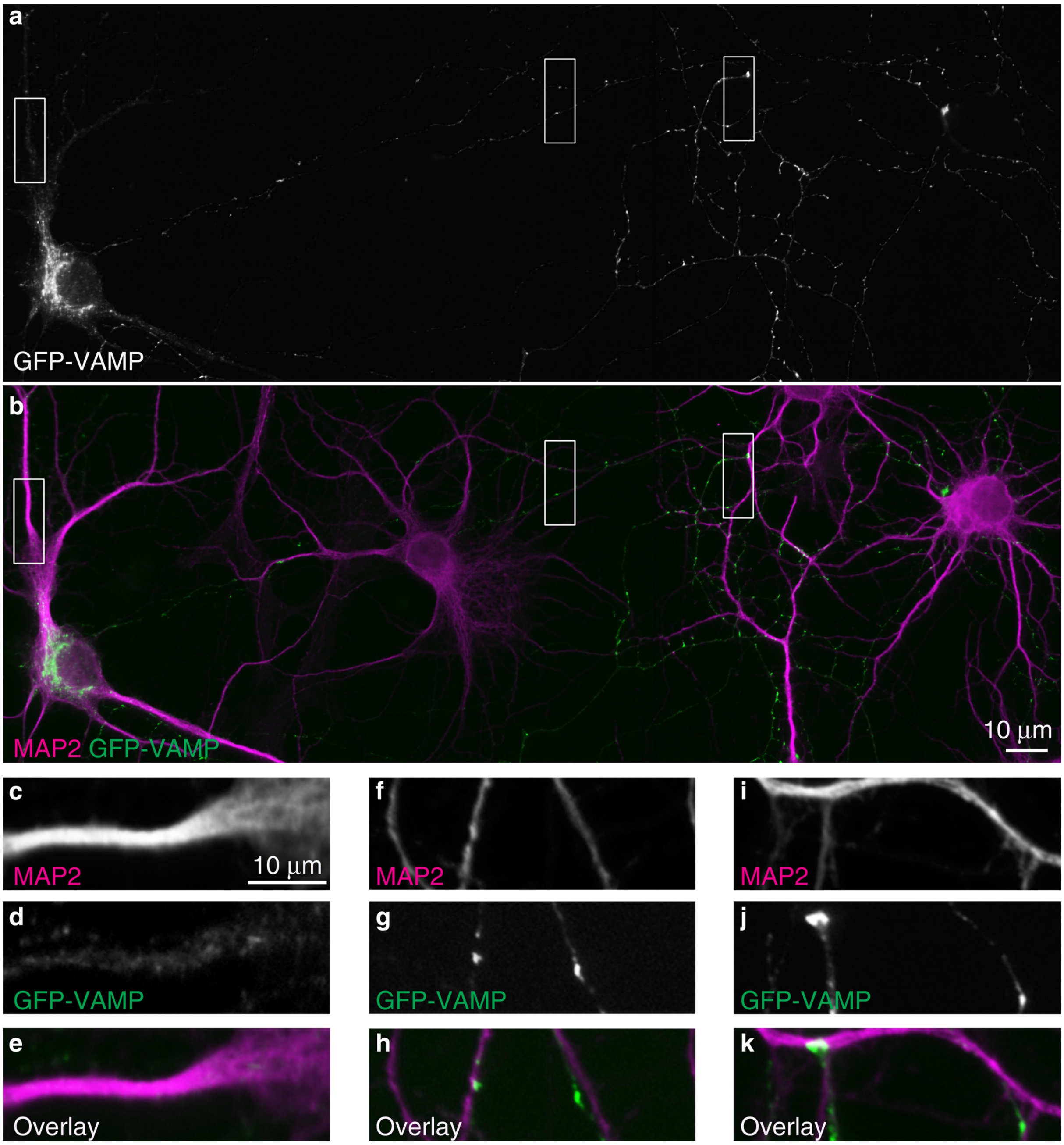

Figure S7:

Example of Presynaptic Targeting of GFP-VAMP in a Transfected Neuron

The experiment shows the distribution of a prototypic synaptic vesicle protein, i.e. GFP-tagged VAMP/Synaptobrevin, in a transfected neuron. GFP-Rogdi (Figure 4 and suppl. figure 6) shows a similar distribution.

(a) Grey level image of GFP-VAMP fluorescence. The neuron on the left is transfected. The image is composed of two overlapping images taken with a 40x objective. The two images were merged because the transfected neuron and its axonal arborisations cannot be

demonstrated in one field of view. GFP-VAMP is detected in the juxtanuclear region of the transfected neuron, weaker fluorescence is detected in the proximal parts of its dendrites, and the strongest fluorescence is punctate in the right part of the field of view. Some of the puncta are located more than 200 µm away from the soma of the transfected neuron. (b) The image shows GFP-VAMP and MAP2. MAP2 staining reveals that in addition to the transfected neuron parts of four untransfected neurons are on the image: two complete cell bodies with dendrites emerging from them are visible; part of a third neuron's cell body is visible at the top right; dendritic arborisation from a fourth neuron extend from the bottom right upwards into the field of view. (a,b) The left box is magnified in c-e, the middle box is magnified in f-h, the right box is magnified in i-k. (c-e) Relatively weak (compared to the juxtanuclear fluorescence and the punctate axonal fluorescence) and diffusely distributed GFP-VAMP fluorescence in a proximal part of a dendrite of the transfected neuron. (f-g) Punctate axonal GFP-VAMP fluorescence in close proximity to the dendrites of an untransfected neuron. (i-k) Another example of the situation depicted in f-h, showing GFP-VAMP puncta in close apposition to dendrites of untransfected neurons.

Supplemental Information to Figure S7:

The power of GFP-VAMP/Synaptobrevin variants to accumulate in presynaptic terminals has been firmly established and used to label presynaptic terminals and to monitor synaptic vesicle recycling in live neurons (see e.g. [6–9]). In the present study GFP-VAMP is included to compare the distribution of GFP-Rogdi to the distribution of an established presynaptic marker.

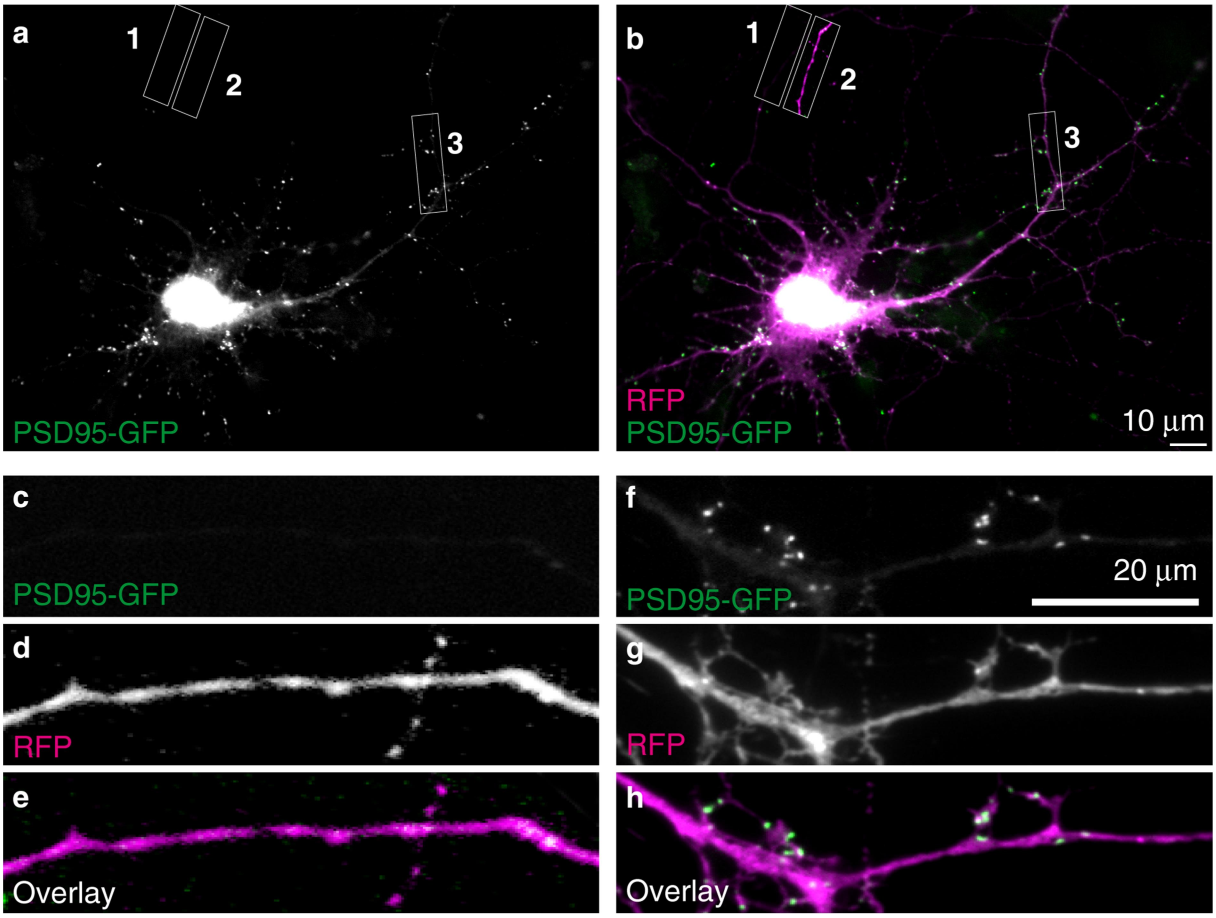

Figure S8:

Example of Postsynaptic Targeting

The experiment shows the distribution of a prototypic postsynaptic protein, i.e. GFP-tagged PSD95, in a transfected neuron. GFP-Rogdi (Figures 4, 5, and suppl. figure 6) and GFP-VAMP (Figure 5 and suppl. figure 7) do not share any resemblance with the distribution of PSD95-GFP.

(a) Grey level image of PSD95-GFP fluorescence in a transfected neuron. PSD95-GFP is punctate along the dendrites of the transfected neuron. The axon is not visible because it does – as expected – not contain any PSD95-GFP. Because the axon of a PSD95-GFP expressing neuron does not contain any GFP fluorescence and therefore is not visible, we co-transfected the neuron with a red fluorescent soluble protein that diffuses into all compartments, i.e. RFP. (b) The image shows an overlay of PSD95-GFP fluorescence and RFP-fluorescence. Box 2 is a brightness-enhanced version of the area outlined by box 1, to

reveal the axonal RFP fluorescence in this low magnification image. (c-h) High magnification images of two regions of the transfected neuron. (c-e). The panels represent PSD95-GFP fluorescence and RFP-fluorescence in box 1. PSD95-GFP is absent in this RFP-positive process, i.e. presumably the axon. (f-h) The panels represent PSD95-GFP fluorescence and RFP-fluorescence in box 3. PSD95-GFP is punctate in the thin protrusions (filopodia or spines) originating from a dendritic branch of the transfected neuron.

Supplemental Information to Figure S8:

The experiment is to demonstrate a typical postsynaptic staining pattern produced by a prototypic postsynaptic protein. It indicates that a postsynaptic staining pattern is characterized by the following features:

1) A punctate distribution of fluorescence in the dendrites of the transfected neuron.

2) The puncta are located relatively close to the cell body because they are in the dendrites of the transfected neuron.

3) The puncta are aligned along the dendrites of the transfected neuron because they represent most or all of the postsynaptic sites of the transfected neuron. Thus, they outline the overall shape of the dendritic arborisation of the transfected cell.

4) Absence of fluorescence in the axon. To demonstrate that an axon is in the field of view, neurons have to be co-transfected with a marker that is found in the axon.

Typical postsynaptic staining patterns, similar to what we present in figure S8, can also be seen in the following studies:

Figure 1 in
https://www.ncbi.nlm.nih.gov/pubmed/?term=dresbach+prosap

Figure 1 in
https://www.ncbi.nlm.nih.gov/pubmed/11520177

Figure 1 in
https://www.ncbi.nlm.nih.gov/pubmed/14960624

The distribution of GFP-Rogdi and GFP-VAMP show opposite characteristics compared to

PSD95-GFP, i.e they are characterized by the following features:

1) Weak and diffusely distributed fluorescence in the dendrites of the transfected neuron. Dendritic fluorescence may be absent or below detection limit in distal parts of dendrites.

2) The puncta are located at long distances from the cell body of the transfected cell.

3) The puncta are irregularly arranged, because they represent presynaptic specialization of the axon, which are formed wherever this axon contacts the dendrites of other neurons.

4) The course of the axon is sometimes visible due to weak background fluorescence between the punctate accumulations, presumably resulting from material being transported to synapses or exchanged between synapses.

Typical presynaptic staining patterns, similar to what we observe for GFP-Rogdi, GFP-VAMP and Synaptophysin-mCherry can also be seen in the following studies:

Figure 1 in
https://www.ncbi.nlm.nih.gov/pubmed/15039456

Figure 2 in
https://www.ncbi.nlm.nih.gov/pubmed/10931840

Figure 1 in
https://www.ncbi.nlm.nih.gov/pubmed/15071120

Figure 7 in
https://www.ncbi.nlm.nih.gov/pubmed/15911881

Figure 2 in
https://www.ncbi.nlm.nih.gov/pubmed/12812759

Figure 12 in
https://www.ncbi.nlm.nih.gov/pubmed/23723986

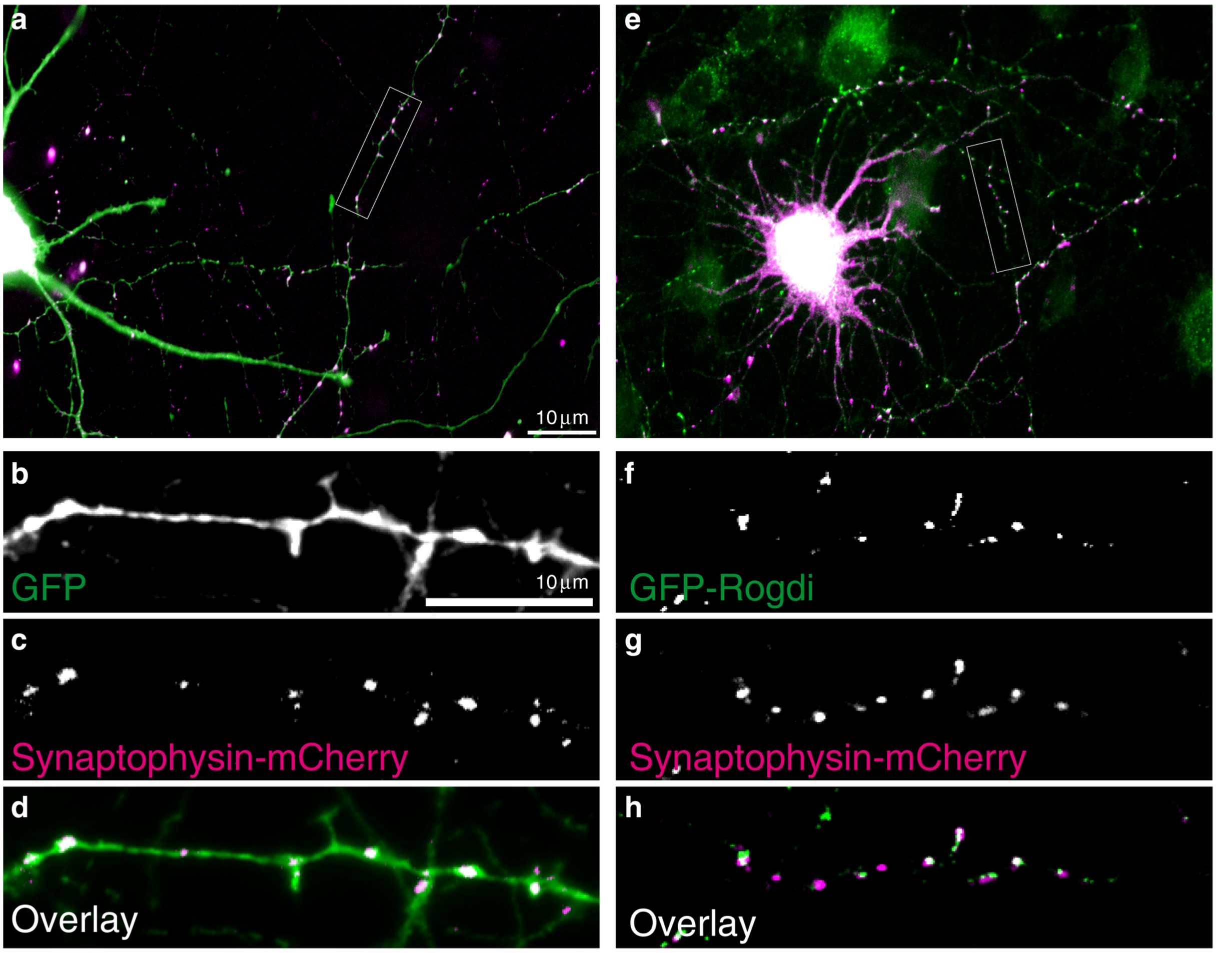

Figure S9:

GFP-Rogdi Colocalises with GFP-Synaptophysin

(a-d) Synaptophysin is a synaptic vesicle protein. Recombinant versions of Synaptophysin are used to label presynaptic boutons in transfected neurons. When we co-transfected neurons with GFP and a red fluorescent variant of Synaptophysin, i.e. Synaptophysin-mCherry, GFP filled the entire neuron homogeneously, while Synaptophysin-mCherry accumulated in axonal hot spots, expected to represent presynaptic boutons. (e-f) When we co-transfected neurons with GFP-Rogdi and Synaptophysin-mCherry, GFP-Rogdi produced a punctate staining pattern that was different from that produced by GFP (as expected from figures 1-7), and showed extensive colocalisation with Synaptophysin-mCherry. Because recombinant Synaptophysin is an established marker for presynaptic boutons, this further corroborates the notion that GFP-Rogdi accumulates at presynaptic sites.

Supplemental Information to Figure S9:

Like fluorescent VAMP/Synaptobrevin constructs, fluorescent Synaptophysin is used to label presynaptic boutons in transfected neurons [10–13]. The colocalisation of GFP-Rogdi with Synaptophysin-mCherry further corroborates the notion that GFP-Rogdi behaves like a presynaptic protein, i.e. it accumulates at presynaptic site.

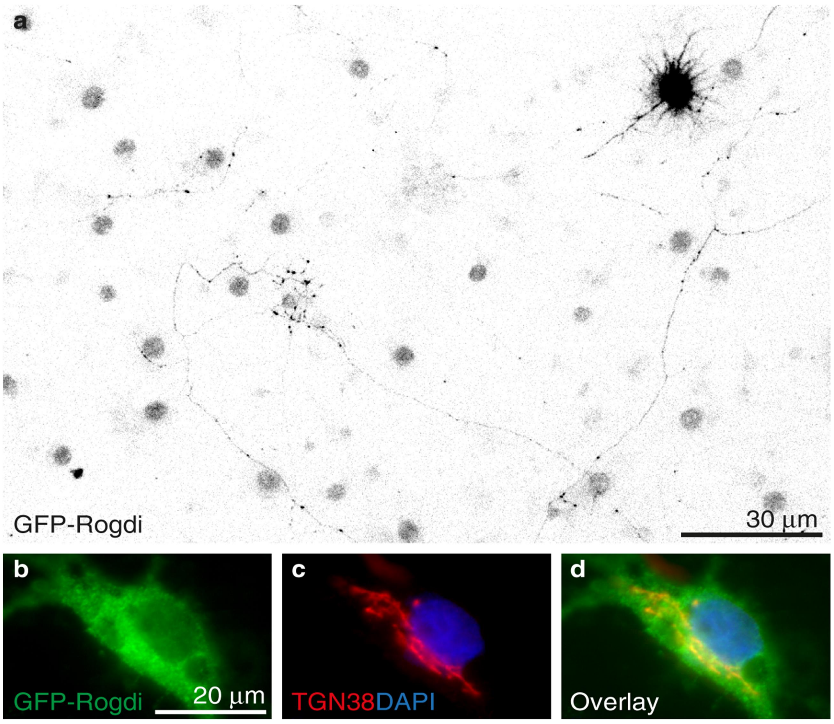

Figure S10:

Representative Examples of the Distribution of GFP-Rogdi in DIV7 neurons

DIV7 represents a stage of culture development where dendrites are still short and synapse formation is before its peak [14]. (a) Grey level image of green fluorescence. The neuron in the upper right is transfected with GFP-Rogdi. Axonal hot spots of GFP-Rogdi fluorescence are visible all along the axon and particularly frequent in the left part of the image. Note that the soma is out of focus. (b-d) Image of a GFP-Rogdi expressing neuron where the cell body and nucleus are in focus. GFP-Rogdi (green) is largely excluded from the nucleus (blue; stained with DAPI) and homogeneously distributed throughout the soma. Because some presynaptic proteins are known to associate with the Golgi-apparatus (e.g. Bassoon [15]) we stained for the Golgi marker TGN38 (red). GFP-Rogdi fluorescence does not obviously associate with the Golgi-apparatus.

References

1. Matus, A., Bernhardt, R. & Hugh-jonest, T. High molecular weight microtubule-associated proteins are preferentially associated with dendritic microtubules in brain. *Proc. Natl. Acad. Sci. U. S. A.* **78,** 3010–3014 (1981).
2. Dotti, G., Sullivan, A., Biology, C., College, A. M. & York, N. The Establishment of Polarity by Hippocampal. *J. Neurosci.* **8,** 1454–1468 (1988).
3. Dresbach, T. *et al.* Functional regions of the presynaptic cytomatrix protein Bassoon : significance for synaptic targeting and cytomatrix anchoring. *Mol. Cell. Neurosci.* **23,** 279–291 (2003).
4. Dean, C. *et al.* Distinct Subsets of Syt-IV/BDNF Vesicles are Sorted to Axons Versus Dendrites and Recruited to Synapses by Activity. *J. Neurosci.* **32,** 5398–5413 (2012).
5. Chen, Y. *et al.* Downregulation of a novel human gene , ROGDI , increases radiosensitivity in cervical cancer cells. *Cancer Biol. Ther.* **17,** 1070–1078 (2016).
6. Alsina, B., Vu, T. & Cohen-cory, S. Visualizing synapse formation in arborizing optic axons in vivo : dynamics and modulation by BDNF. *Nat. Neurosci.* **4,** 1093–1101 (2001).
7. Hu, B., Nikolakopoulou, A. M. & Cohen-cory, S. BDNF stabilizes synapses and maintains the structural complexity of optic axons in vivo. *Development* **132,** 4285–4298 (2005).
8. Li, Z. *et al.* Synaptic vesicle recycling studied in transgenic mice expressing synaptopHluorin. *Proc. Natl. Acad. Sci. U. S. A.* **2005,** 6131–6136 (2005).
9. Stan, A. *et al.* Essential cooperation of N-cadherin and neuroligin-1 in the transsynaptic control of vesicle accumulation. *Proc. Natl. Acad. Sci. U. S. A.* **107,** 11116–11121 (2010).
10. Dresbach, T. *et al.* Membrane Transport, Structure, Funtion, and Biogenesis: Assembly of Active Zone Precursor Vesicles: Obligatory trafficking of presynaptic cytomatrix proteins Bassoon and Piccole via a trans-Golgi compartment. *J. Biol. Chem.* **281,** (2006).

11. Sun, Y. & Bamji, S. X. □ -Pix Modulates Actin-Mediated Recruitment of Synaptic Vesicles to Synapses. *J. Neurosci.* **31,** 17123–17133 (2011).
12. Bury, L. A. D. & Sabo, S. L. Coordinated trafficking of synaptic vesicle and active zone proteins prior to synapse formation. *Neural Dev.* **6,** 24 (2011).
13. Kwon, S. E. & Chapman, E. R. Synaptophysin regulates the kinetics of synaptic vesicle endocytosis in central neurons. *Neuron* **70,** 847–854 (2011).
14. Kaech, S. & Banker, G. Culturing hippocampal neurons. *Nat. Protoc.* **1,** 2406–2415 (2006).
15. Dresbach, T. *et al.* Assembly of Active Zone Precursor Vesicles: obligatory trafficking of presynaptic cytomatrix proteins Bassoon and Piccolo via a trans-Golgi compartment. *J. Biol. Chem.* **281,** 6038–6047 (2006).
